# Diversity of Cultivated Fungi Associated with Conventional and Transgenic Sugarcane and the Interaction between Endophytic *Trichoderma virens* and the Host Plant

**DOI:** 10.1371/journal.pone.0158974

**Published:** 2016-07-14

**Authors:** Aline Silva Romão-Dumaresq, Manuella Nóbrega Dourado, Léia Cecilia de Lima Fávaro, Rodrigo Mendes, Anderson Ferreira, Welington Luiz Araújo

**Affiliations:** 1 Department of Genetics, Escola Superior de Agricultura “Luiz de Queiroz”(ESALQ), University of São Paulo, São Paulo, Brazil; 2 Laboratory of Molecular Biology and Microbial Ecology, Department of Microbiology, Institute of Biomedical Sciences, University of São Paulo, São Paulo, Brazil; 3 Brazilian Agricultural Research Corporation, Embrapa Agroenergy, Brasília, Distrito Federal, Brazil; 4 Brazilian Agricultural Research Corporation, Embrapa Environment, Jaguariuna, São Paulo, Brazil; 5 Brazilian Agricultural Research Corporation, Embrapa Agrosilvopastoral, Sinop, Mato Grosso, Brazil; University of Calcutta, INDIA

## Abstract

Plant-associated fungi are considered a vast source for biotechnological processes whose potential has been poorly explored. The interactions and diversity of sugarcane, one of the most important crops in Brazil, have been rarely studied, mainly concerning fungal communities and their interactions with transgenic plants. Taking this into consideration, the purpose of this study was, based on culture dependent strategy, to determine the structure and diversity of the fungal community (root endophytes and rhizosphere) associated with two varieties of sugarcane, a non-genetically modified (SP80-1842) variety and its genetically modified counterpart (IMI-1, expressing imazapyr herbicide resistance). For this, the sugarcane varieties were evaluated in three sampling times (3, 10 and 17 months after planting) under two crop management (weeding and herbicide treatments). In addition, a strain of *Trichoderma virens*, an endophyte isolated from sugarcane with great potential as a biological control, growth promotion and enzyme production agent, was selected for the fungal-plant interaction assays. The results of the isolation, characterization and evaluation of fungal community changes showed that the sugarcane fungal community is composed of at least 35 different genera, mostly in the phylum Ascomycota. Many genera are observed at very low frequencies among a few most abundant genera, some of which were isolated from specific plant sites (e.g., the roots or the rhizosphere). An assessment of the possible effects upon the fungal community showed that the plant growth stage was the only factor that significantly affected the community’s structure. Moreover, if transgenic effects are present, they may be minor compared to other natural sources of variation. The results of interaction studies using the Green fluorescent protein (GFP)-expressing *T*. *virens* strain *T*.*v*.223 revealed that this fungus did not promote any phenotypic changes in the host plant and was found mostly in the roots where it formed a dense mycelial cover and was able to penetrate the intercellular spaces of the root epidermis upper layers. The ability of *T*. *virens* to colonize plant roots suggests a potential for protecting plant health, inhibiting pathogens or inducing systemic resistance.

## Introduction

Sugarcane (*Saccharum* sp.) is an important crop in Brazil, mainly for the production of ethanol, a biofuel, as a renewable energy source that is already an alternative to petrol in Brazilian cars. To increase productivity and decrease costs, researchers are investing in new sugarcane varieties developed by classical breeding as well as genetically modified plants, searching for characteristics such as insect resistance, herbicide resistance, a higher sugar content, etc [1, 2]. Therefore, studies on biosecurity and environmental impact should be performed before releasing the genetically modified plants for commercial production

The microbial community of the host plant can have a positive, negative or neutral effect on the plant, caused mainly by those microorganisms that live in intimate contact with the plant tissues, such as endophytes or rhizoplane microbes [3]. Studies reported the effects of the genetically modified plant on the microbial community associated [4–8], but these effects were minor in comparison with the effect caused by cultivar [5,9], soil type [10,11], crop location [12,13] and plant growth stage [5, 14, 15, 16, 17].

Microbial diversity studies can help elucidate the role of native microorganisms in the ecosystem and the association of biological changes with microbial diversity [18,19]. A few studies have been performed with transgenic sugarcane, mainly involving beneficial fungi and plant interactions [20,21]. Fungi play a key role in several ecological processes essential for ecosystem maintenance [22]. Endophytic fungi colonize plant tissue internally without causing damage to the plant host [23], establishing a balanced and mutualistic interaction [24]. The fungus receives nutrients while the plant benefit from the inhibition of pathogens and stress resistance as well as increased growth [25].

On the other hand, the rhizosphere is influenced by root exudates and is intensely colonized by microbial communities [26], which have direct effects on plant growth and nutrient availability or protecting against pathogens [27, 28]. Several rhizosphere fungi degrade toxic compounds, such as xenobiotic and aromatic molecules, and are essential for the survival of plants in contaminated soils [29]. Other fungi, such as *Fusarium oxysporum*, a nonpathogenic strain, can suppress pathogenic strains [30]. Many fungi, such as members of the *Trichoderma* genus, can inhibit phytopathogens and act as a biological control [31]. Among these, *Trichoderma virens* has been reported to be aggressive mycoparasite [32, 33], able to parasitize not only hyphae but also fungal resistance structures [32, 33, 34]. In addition to its mycoparasitic activity, it can also produce extracellular chitinase [35] and several antibiotics and can induce the production of plant fitoalexins [36]. *T*. *virens* has been considered a versatile and effective biological control agent. Moreover, *T*. *virens* has been reported to be a plant endophyte [37], able to asymptomatically colonize the host plant and occupy the same niche as phytopathogens. In addition, *Trichoderma* spp. strains have been used to promote plant growth [31,38,39] and show a great potential for agricultural use.

In the present study we considered the hypothesis that if the genetically modified plant could have an impact on the microbial communities associated, the accumulation of these effects over the time could be detected after a long period of the plant in the field. Therefore, we investigated the effect of genetically modified sugarcane, sampling time (plant growth stage) and crop management (weeding and herbicide application) on the structure and diversity of the cultivable fungus communities associated with sugarcane plants. Rhizosphere and root endophytic fungi were isolated from two sugarcane varieties, a non-transgenic (SP80-1842) and a genetically modified (IMI-1, expressing imazapyr herbicide resistance) variety, treated and untreated with the herbicide imazapyr, at three different growth stages over a two-year period. *Trichoderma*, a genus abundant in sugarcane, which has potential agricultural use, was selected for these plant interaction studies.

## Material and Methods

The sugarcane orchard was not in a protected area. Sugarcane plants (*Saccharum officinarum*) were grown in the experimental area of the Centro de Tecnologia Canavieira S.A. (CTC), a private company that present the CQB (Certificate for Biosecurity Quality) for the cultivation area, in Piracicaba, São Paulo, Brazil (22° 41’ S e 47° 33’ O), under supervision of Dr. Eugenio Cesar Ulian a Director of the CTC company. Therefore, the only one permission that we need for this experiment permission was gave by the experimental Company Supervisor, Dr. Eugenio Cesar Ulian."

### Sugarcane experiments

Sugarcane plants (*Saccharum officinarum*) were grown in the experimental area of the Centro de Tecnologia Canavieira S.A. (CTC), Piracicaba, São Paulo, Brazil (22° 41’ S e 47° 33’ O) under supervision of Dr. Eugenio Cesar Ulian. We used the transgenic sugarcane IMI-1, which expresses resistance to the herbicide imazapyr, and the non-transgenic isoline cultivar SP80-1842. Three treatments were used in a randomized experimental design with four replicates: i) the non-transgenic sugarcane SP80-1842 + weeding (NW), ii) the transgenic sugarcane IMI-1 + weeding (TW) and iii) the transgenic sugarcane IMI-1 with imazapyr herbicide applied two months after planting (TH). In this experimental design the TW x TH comparison allowed evaluate the crop management (weeding x herbicide application) and NW x TW comparison allowed evaluate the plant genotype (non-transgenic X genetically modified variety). Samples were collected at 3, 10 and 17 months after planting (sampling times), and each sample was comprised by four replicates (with 3 plants each).

### Isolation of sugarcane endophytic and rhizosphere fungi

Endophytic and rhizosphere fungi were isolated from sugarcane according to Stuart et al. [20]. For endophytic fungi isolation, roots were surface sterilized and for each three treatments (NW, TW and TH), the roots of three plants (from each replicate) were cut in 21 fragments (5 mm x 5mm each) and transferred onto Potato Dextrose Agar (PDA, Merck, Kenilworth, NJ, USA) with tetracycline (50 μg mL^-1^) to inhibit bacterial growth. For rhizosphere fungi isolation, 5 g of rhizosphere soil was diluted in 50 mL of PBS buffer (NaCl 140 mM, KCl 3 mM, Na_2_HPO_4_ 10 mM and KH_2_PO_4_ 2 mM, pH = 7.4) and shaken with glass beads for 1 h at 150 rpm. The suspension was diluted and the 10^−3^ and 10^−1^ dilutions were plated on PDA with tetracycline as used in previous study (Stuart et al., 2010). The plates were incubated at 28°C for 2 to 14 days. After fungal growth, the colonies were counted and grouped by morphological characteristics. Filamentous fungal isolates were purified by serial dilution and stored. The frequency of the colony grown per fragment and per soil gram was calculated and converted to the number of colony forming units per gram (CFU g^-1^).

### DNA extraction and molecular identification of the endophytic and rhizosphere fungi

DNA extraction of the isolated fungi was performed according to Raeder & Broda [40] with modifications. The fungi were grown in 50 mL of PDB (Potato Dextrose Broth) for 5 days at 28°C. The fungal suspension was filtered, and the mycelia were ground in liquid nitrogen. The grounded mycelia (200 mg) were transferred to 1 mL of extraction buffer (SDS 1%, EDTA 25 mM, NaCl 250 mM and Tris-HCl 200 mM [pH 8,0]), incubated at 65°C for 20 min and centrifuged at 16,000 X *g* for 10 min at 4°C. The DNA was purified using phenol/chloroform and precipitated with isopropanol. After the DNA was extracted, the DNA was quantified and its integrity was assessed by electrophoresis in a 0.8% of agarose gel; the DNA was stored at -20°C.

The ITS1-5.8S-ITS2 region of rDNA was amplified using ITS-1 and ITS-4 primers [41]. After electrophoresis, the fragments were purified and sequenced at the Centro de Estudos do Genoma Humano (University of São Paulo, São Paulo, Brazil). To identify the fungal isolates, the sequences were compared to the GenBank database via BLASTn (http://www.ncbi.nlm.nih.gov/BLAST). Partial DNA sequences of the ITS1-5.8S-ITS2 region were deposited in the GenBank database under the accession numbers GQ495269 and GU973612-GU973859.

### Sequence analysis and phylogeny

All the chromatograms were first trimmed for high quality bases (80% of bases with quality > 20) by means of Phred software, and the trimmed sequences were used for comparison in the GenBank database (nr/nt). The best hits of well-characterized strains were retrieved from the databases and subsequently used for alignment and phylogeny analysis with MEGA 4.0 version software [42]. The evolutionary history was inferred through the Neighbor-Joining method [43] and evolutionary distances were computed using the Jukes-Cantor method.

### Determination of endophytic and rhizosphere fungal community structures

The diversity and richness of the fungal community in the three treatments (NW, TW and TH) for three isolation periods (3, 10 and 17 months) were quantified using the Shannon-Wienner (*H’*) and Chao1 index, respectively. These indices were calculated using the DOTUR program [44], which also generated rarefaction data used to infer coverage considering the different similarity levels of the ITS1-5.8S-ITS2 sequence of fungus rDNA. The similarity matrix was generated using the DOTUR program, aligning the sequence and calculating the distance matrix using the Phylip DNADist program (http://bioweb.pasteur.fr/seqanal/interfaces/dnadist-simple.html). The taxonomic levels were calculated with the following cutoffs: 100% (strains), 99% (species) and 97%-95% (genera). The similarity coefficient of Jaccard (J) was calculated, considering the generic level.

AMOVA was performed to estimate the genetic variation among the different fungal communities and to determinate how much of the genetic variation could be attributed to the differences among the sugarcane genotypes (conventional vs. transgenic), crop management (weeding vs. imazapyr), the sampling period (growth stages) and the rhizosphere and endophytic fungal community. All analyses of the DNA sequences from the fungal isolates were performed using the Arlequin program version 3.1 [45], considering 16.000 permutations.

A Principal Component Analysis (PCA) was used to verify whether a correlation existed between the fungal communities found in the rhizosphere and those found in the endosphere. The PCA analysis was performed using *Canoco version 4*.*5* [46], using the frequency of each genus in each repetition of endophytic and rhizosphere samples as the input variable.

### *Agrobacterium*-mediated transformation

The isolate *T*. *virens* strain *T*.*v*.223 (GenBank access number GQ495269) obtained from inner root tissues was selected to further studies. For this, transformation was carried out according to a previously described method [47] with modifications [21, 48]. Briefly, *A*. *tumefaciens* EHA105 transformed with the plasmid pFAT-*gfp* [49] was grown at 28°C for 16–20 h. The culture suspension was diluted to a λ_660_ = 0.15 in an induction medium broth (10 mM K_2_HPO_4_; 10 mM KH_2_PO_4_; 2.5 mM NaCl; 2 mM MgSO_4_; 0.7 mM CaCl_2_; 9 mM FeSO_4_; 4 mM NH_4_SO_4_; 10 mM glucose; 0.5% glycerol; 40 mM 2-morpholinoethanesulfonic acid, pH 5.3) supplemented with acetosyringone (200 mM) and incubated until a λ_660_ = 0.6 was reached. The cell suspension was mixed 1:1 with a conidial suspension (10^6^ or 10^7^ conidia. mL^-1^) from *T*. *virens* strain *T*.*v*.223. Aliquots were spread on different types of membranes (cellophane, filter paper, nitrocellulose, or nylon) on induction medium agar (5 mM glucose instead of 10 mM) supplemented with 200 mM or 400 mM of acetosyringone. After co-cultivation at 25°C for 24 h or 48 h, the membranes were transferred onto PDA or M-100 [50] media amended with hygromycin B (200 μg mL^–1^; to select transformants) and cefotaxime (200 μg mL^–1^; to eliminate the bacteria).

Individual transformants were subsequently transferred to PDA supplemented with 200 μg mL^-1^ hygromycin. The mitotic stability of the integrated T-DNA was tested by sub culturing five-fold randomly selected transformants on PDA supplemented with 200 μg mL^-1^ of hygromycin incubated at 28°C for 5 days.

### Molecular and morphological characterization of transformants

The genomic DNA of several selected transformants was extracted according to Raeder and Broda [40], and the presence of the *gfp* gene that express the green fluorescent protein was verified by PCR. We used the primers glGFP5 (5’-GCCGGAATTCATGAGCAAGGGCGAGGAACTGTTC-3’) and glGFP3 (5’-GCCGAGCTCAGATCTCACTTGTACAGCTCGTCCATGCC-3’) that amplified 700 pb of *gfp* gene [49].

To check the number of inserts in the genome, four transformants (T2, T7, T10 and T20) and the wild type strain (used as a negative control) were analyzed by Southern blotting. The DNA (15 μg) of each strain was cleaved with 10 U μL^-1^ *Eco*RI (Invitrogen, Life Technologies) incubated at 37°C for 12 h [49]. The digested DNA was electrophoresed in a 1% agarose gel. The probe used in the Southern blot analysis, the 700 pb sequence of *gfp* gene, was labeled using a Gene Images^™^ AlkPhos Direct^™^ Labelling and Detection System (GE Healthcare) kit according to the manufacturer’s protocol.

The gel was washed with a 0.25 M HCl solution for 10 min, followed by distilled water, a denaturing solution (NaOH 0.5 M, NaCl 1.5 M) for 30 minutes, distilled water, and two washes with a neutralization solution (Tris-HCl 0.5 M; NaCl 1.5 M; EDTA 0.001 M [pH = 7.2]). The DNA in the gel was transferred to a nylon membrane (Hybond N^+^, Amersham) for 12 h in transference solution SSC 20X (NaCl 3 M; sodium citrate 0.3 M [pH = 7.0]). The membrane was dried for 2 h at 80°C and stored at 4°C.

Morphological characterization was performed to compare the wild type and mutant strains on the basis of growth rate and morphology (sporulation rate and color). The strains were grown for 5 days in PDA at 28°C, and the colony diameter was measured after 24, 48, 72 and 96 h. All tests were performed in triplicate.

### *Trichoderma virens–*host plant interaction

#### Sugarcane plant reisolation of *Trichoderma virens* mutant T20

The mutant T20, which expressed the *gfp* gene and hygromycin resistance, was grown in PDA (Merck) at 28°C for 7 days to obtain conidia. *In vitro* sugarcane plants (variety SP80-1842) from Centro de Tecnologia Canavieira (CTC, Piracicaba, SP, Brazil), were transferred to glass flasks containing 20 mL of MS liquid broth [51] with 10^6^ conidia mL^-1^ of mutant T20. The plants were incubated at 28°C under a 16 h light and 8 h dark cycle. After 48 h, the plants were transferred to pots containing a commercial substrate PlantMax (Eucatex) and placed in a greenhouse to acclimatize. Non-inoculated sugarcane plants (variety SP80-1842) were similarly treated as a control. After 20, 40 and 60 days in the greenhouse, the sugarcane plants were sampled and leaves, stem and roots were surface-disinfected according to Stuart et al. [20]. After processing, the leaf and stem tissues were cut in fragments of approximately 100 mm^2^ (10 mm x 10 mm) and roots were cut in 5 mm fragments. A total of 1512 plant fragments (504 fragment per sampling time) were transferred to PDA (Merck) with tetracycline (100 μg mL^-1^) to inhibit bacterial growth. Hygromycin B (200 μg mL^-1^) was also added to the culture medium for the T20 mutant. The isolation plates were incubated at 28°C and periodically evaluated up to 14 days. After growth, the total fungal colonies and *Trichoderma* colonies were counted, and *T*. *virens* strain T20 were confirmed by *gfp* fluorescence and hygromycin B resistance.

#### Microscopic analysis of *Trichoderma virens* and sugarcane interaction

Axenic sugarcane plants (variety SP80-1842) were inoculated with *T*. *virens strain* **T20** conidia (with the *gfp* gene) and incubated at 28°C under a 16 h light and 8 h dark cycle. After 24, 48, 72 and 168 h, the roots were processed according to Ferreira et al. [52] and observed under an optical microscope (Zeiss Axiophot-2) using two filters, FITC (459–490 nm) and Rhodamine (546 nm), to differentiate sugarcane fluorescence from GFP fungus fluorescence. Images were captured separately with each filter and then overlapped using the ISIS software (Meta Systems, Germany).

The same roots were collected after 12, 24, 48 and 72 h, and fixed with Karnowsky solution and processed according to Rossetto et al. [53]. In addition to the colonization of the root surface, the inner root tissues were visualized via a freeze-fracture technique by fixing the sample with Karnowsky solution and washing with 0.05 M cacodylate buffer. The roots were treated with 30% glycerol for 2 h, wrapped in tissue paper and frozen in liquid nitrogen. The roots were fractured and dehydrated in ethanol, subjected to critical point drying and coated with metal. The samples were observed in a scanning electron microscope (LEO-Zeiss) at the Núcleo de Apoio à Pesquisa em Microscopia Eletrônica (NAP/MEPA), University of São Paulo.

#### *Trichoderma virens–*plant interaction assessment

The effect of the *T*. *virens* wild type strain (*T*.*v*.223) and a strain containing *gfp* (T20**)** on sugarcane (variety SP80-1842) growth were measured. The plants were inoculated with 10^6^ conidia mL^-1^ of the wild type strain (*T*.*v*.223) or the *gfp-*tagged strain (T20**)** in a commercial PlantMax substrate (Eucatex). Non-inoculated plants were used as a control and were maintained in a greenhouse for two months, after which the plant fresh and dry mass were determined. A total of 23 plants per treatment were used in a completely randomized experimental design. To confirm fungal colonization, *T*. *virens* was reisolated from the inoculated plants as previously described.

### Statistical analysis

The solation data were statistically analyzed using the fungus frequency (Ff = number of fungus colony/number of root fragments) for the endophytic fungal community and the fungus density (Df = number of forming unit colony/gram of soil) for the rhizosphere fungal community. An analysis of variance (ANOVA) was performed with *SAS (Copyright (c) 1989–1996 by SAS Institute Inc*., *Cary*, *NC*, *USA)* for a completely randomized experimental design.

## Results

### Isolation of fungi associated with sugarcane

To analyze the potential effects of genetically modified sugarcane on fungal frequency (Ef) (in root endophyte) and density (Df) (in rhizosphere), we isolated fungi from the rhizosphere and endorhizosphere of genetically modified plants that expressed an imazapyr resistance gene (IMI-1) and the conventional isoline (SP80-1842) for three different treatments (NW, TW and TH) at three growth stages of sugarcane plants (3, 10 and 17 months).

**Fig 1:**
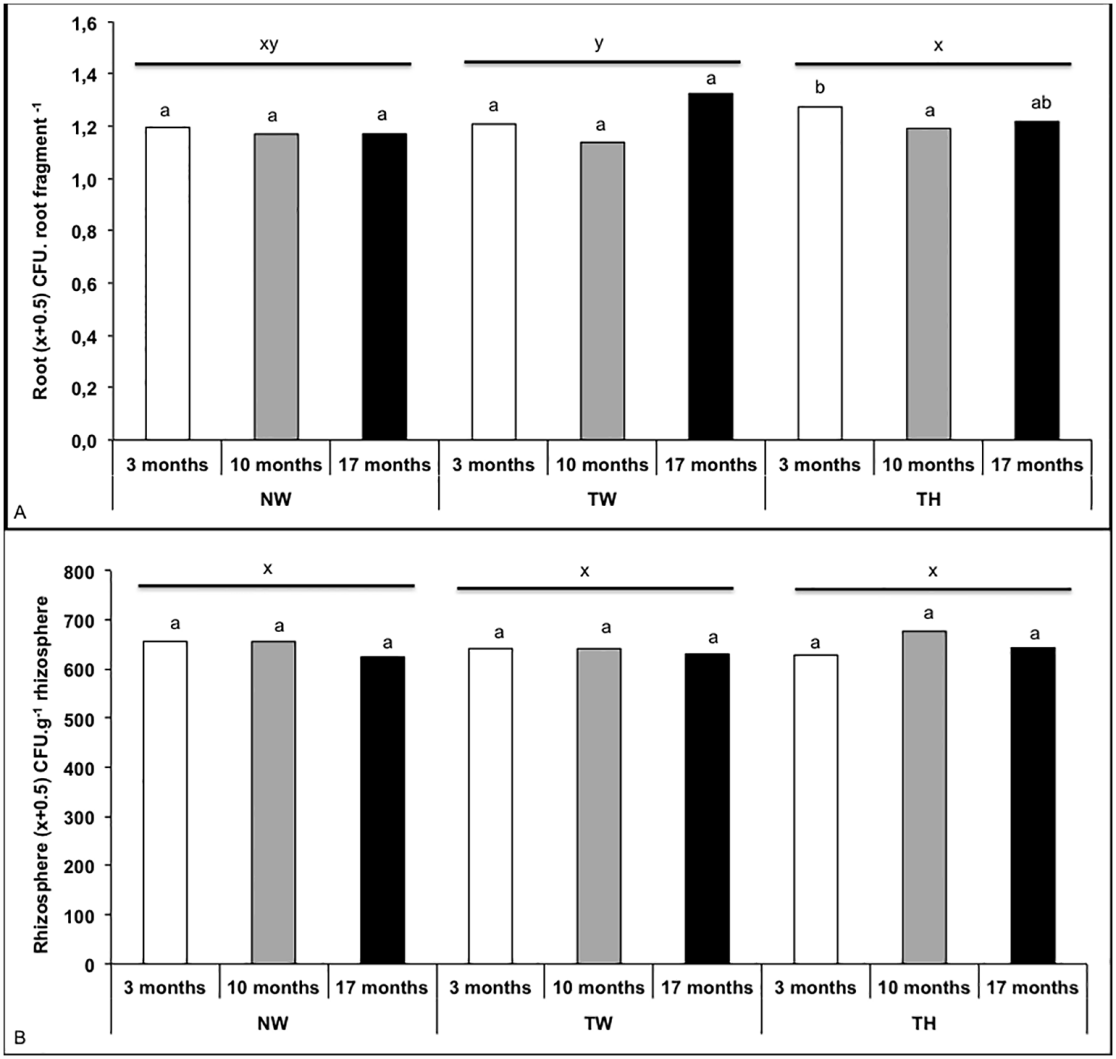
Filamentous fungal isolation of root entophytes frequency (a) and rhizosphere density (b). The bars indicate the average of each effect in each variation factor: treatments (NW**-** non-transgenic plants with weeding, TW- genetically modified plants with weeding and TH**-** genetically modified plants treated with herbicide); sampling time (3, 10 and 17 months after planting). Different letters represent difference at 5% probability in the Tukey test. Comparison among sampling time (3, 10 and 17 months) we used letter a and b and comparison of treatments (NW, TW, TH) we used letters x and y.

The experiment evaluated the effects of plant genotype, crop management and growth stage on the fungal community associated with sugarcane. Sugarcane roots had a high number of endophytic fungi, and from 756 root fragments (252 fragment per sampling time), 2,236 fungal colonies we isolated, being 781 from 3month-old plants, 659 from 10 month old plants and 796 from 17-month-old plants (Fig 1). Similarly, the plant rhizosphere was also found to be highly colonized by fungi, being isolated 1,224 fungi colonies from this plant site. The number of rhizosphere fungi ranged from 4.25 10^5^ CFU g^-1^ of soil at 17 month-old plants to 8.20 10^5^ CFU g^-1^ of soil at 10 month-old plants (Fig 1). The ANOVA for the different treatments (NW, TW and TH) and sampling time (3, 10 and 17 months) was performed on the fungus frequency (endophytic communities) and density (rhizosphere communities) data (S1 Table).

The ANOVA results showed that regardless the fungal community evaluated (root endophyte or rhizosphere), the genetically modified sugarcane did not induce a significant change (P>0.05) in the fungal frequency or density (S1 Table), suggesting that neither the evaluated plant genotype nor crop management had a significant effect on the sugarcane fungal community. The frequency of root endophytic fungi was significantly (P = 0.0256) affected by sampling time (S1 Table), being the frequency 17 month-old > 10-month-old > 3-month-old sugarcane plants (Fig 1). Curiously, such growth effects were not observed in the density of the rhizosphere community (P>0.05) (S1 Table). Additionally, the ANOVA revealed the presence of an interaction (P = 0.0305) between treatment and sampling time for endophytic fungi, so the effect of the different treatments on the fungal frequency could depended on the plant growth stage.

### Composition and distribution of the fungal community associated with sugarcane

Due to the large number of isolates obtained, the fungi were grouped according to morphological characteristics (type of mycelium, color, presence of spores), and representative samples were selected for subsequent analysis. The morphological diversity of fungus associated with sugarcane displayed a great variation in mycelial color, including white, beige, yellow, green, pink, lilac, brown, gray and black (S1 Fig). In addition to the variety of colors, the fungus also differed in mycelia growth habits (cottony, submerged aerial hyphae), the presence of reproductive structures (spores), the growth rate and the secretion of compounds that changed the color of the growth medium.

Therefore, a total of 249 fungi comprising 16 morphotypes (S1 Fig) from all three treatments were selected for molecular identification by sequencing of the ITS1-5.8S-ITS2 region of rDNA. The sequences obtained were used to evaluate the effects of genotype, management and growth stage using a diversity and richness index. The fungal isolates were taxonomically identified at the genus level via BLASTn by comparison with the ITS1-5.8S-ITS2 rDNA sequences available in GenBank and confirmed by morphological characteristics.

Analysis of the ITS1-5.8S-ITS2 sequences showed that the fungal community associated with sugarcane comprised at least 35 different genera (S2 Table). Overall, the phylum Ascomycote predominated among the fungi identified (96.0%), whereas the phyla Zygomycota/Mucoromycotina and Basidiomycota represented only 2.4% and 1.6% of the community, respectively. In the phylum Ascomycote, Eurotiomycetes (43.0%), the classes Sordariomycetes (38.5%) and Dothydeomycetes (7.6%) were predominant, and the most frequent genera were *Penicillium* (33.3%), *Fusarium* (16.9%), *Aspergillus* (7.2%) and *Trichoderma* (4.4%) (S2 Table).

Other genera were reported at a low frequency (<2.8%), and 16 (*Acremonium*, *Alternaria*, *Cladophialophora*, *Colletotrichum*, *Curvularia*, *Exophiala*, *Mariannaea*, *Myrmecridium*, *Myrothecium*, *Paecilomyces*, *Paraphaeosphaeria*, *Phoma*, *Phomopsis*, *Pyricularia*, *Saccharicola* and *Sagenomella*) were represented by only one isolate.

The genus *Penicillium* (teleomorphs *Eupenicillium* or *Talaromyces*), represented 33.3% of the fungi isolated from both the roots and the rhizosphere. A phylogenetic analysis indicated the similarity of 17 different species (*P*. *restrictum*, *E*. *katangense*, *P*. *janthinellum*, *T*. *flavus*, *E*. *javanicum*, *P*. *ochrochloron*, *E*. *reticulisporum*, *E*. *brefeldianum*, *E*. *levitum*, *P*. *vinaceum*, *P*. *radicum*, *P*. *minioluteum*, *T*. *udagawae*, *T*. *trachyspermus*, *P*. *pinophilum*, *T*. *indigoticus* and *P*. *marneffei*), and fourteen isolates were classified as *T*. *trachyspermus*; three isolates were classified as *P*. *pinophilum* and two isolates were classified as *E*. *javanicum* (S2A Fig).

The genus *Fusarium* (teleomorph *Gibberella*) was the second most frequent genus and included 16.9% of the isolates, and five different species were highly similar (*F*. *oxysporum*, *F*. *acutatum*, *F*. *solani*, *F*. *dlaminii* and *G*. *moniliformis*). In this group, 19 isolates were classified as *F*. *oxysporum*, and the remaining 23 isolates were classified only at the genus level (S2B Fig).

The third most frequent genus, with 7.2% of the isolates, was *Aspergillus*, which had 7 different species that were highly similar (*A*. *brasiliensis*, *A*. *flavus*, *A*. *oryzae*, *A*. *versicolor*, *A*. *niger*, *A*. *terreus* and *A*. *fumigatus*). The phylogenetic analysis showed that six isolates were classified as *A*. *brasiliensis;* three isolates were identified as *A*. *terreus* and one isolate was identified as *A*. *fumigatus* (S2C Fig).

The genera *Trichoderma* and *Epicoccum* occurred less frequently than other genera. *Trichoderma* (teleomorph *Hypocrea*), the fourth most frequent genus, was represented by 11 isolates corresponding to 4.4% of fungus community. The phylogenetic analysis allowed the classification of two isolates as *H*. *virens*, three isolates as *T*. *asperellum* and six isolates as *Trichoderma* sp. (S2D Fig). The genus *Epicoccum* was the fifth most abundant genus and represented 3.6% of the total isolates.

In addition to the most frequent genera (*Penicillium*, *Fusarium*, *Aspergillus*, *Trichoderma* and *Epicoccum*), the fungal community associated with sugarcane included 28 other genera in the phylum Ascomycota, the genus *Resinicium* (with 3 isolates) in the phylum Basidiomycota and the genus *Cunninghamella* (with 3 isolates) in the phylum Zigomycota/Mucoromycotina.

In the phylum Ascomycota, the class Sordariomycetes comprised a large number of the isolates (96), with at least 17 genera identified (*Bionectria*, *Chaetomium*, *Chaetosphaeria*, *Colletotrichum*, *Diaporthe*, *Fusarium*, *Mariannaea*, *Microdochium*, *Myrmecridium*, *Myrothecium*, *Nigrospora*, *Paecilomyces*, *Phomopsis*, *Pyricularia*, *Thielavia*, *Thozetella* and *Trichoderma*). Moreover, nine isolates were not similar to any reported genus, so they were classified as *not identified* (N.I.) (S2 Table).

In the phylum Ascomycota, besides the class Sordariomycetes, the fungal community associated with sugarcane was characterized by isolates from the classes Dothideomycetes, Eurotiomycetes, Leotiomycetos and mitosporic fungi. The class Dothideomycetes comprised 19 isolates, classified in six different genera (*Alternaria*, *Cladosporium*, *Curvularia*, *Epicoccum*, *Paraphaeosphaeria* and *Saccharicola*). The Eurotiomycetos class comprised 107 isolates; however, 94.4% were classified in the genera *Aspergillus* and *Penicillium*, and the other 6 isolates were identified as *Cladophialophora*, *Exophiala*, and *Sagenomella*. The class Leotiomycetes had only two representatives, both in the genus *Acephala*.

### Sugarcane fungal community structure

Rarefaction curves were constructed to verify whether the sampling of the present work was adequate to assess the population diversity. Overall, the rarefaction curves of different populations using similarity levels of 95% and 97% were sufficient to encompass the diversity of fungal population (S3 Fig). Therefore, richness and diversity comparisons based on 95% and 97% similarities were performed.

The diversity (Shannon-Wienner) and richness (Chao1) index for the fungal population associated with sugarcane are presented in Table 1. The results show that the fungal community associated with sugarcane has great richness and diversity; the total diversity and richness indices (*H’*_(95%)_ = 3.53; *H’*_(97%)_ = 3.64; *Chao1*_(95%)_ = 98; *Chao1*_(97%)_ = 134) are considered high for the similarity index used. Moreover, rhizosphere seems to have a higher fungal diversity than root endophytes, although the values were not significantly different (Table 1).

**Table 1:** Diversity and Richness index of all sequence groups at 97% and 95% similarity.

Index	Sequence group	Similarity	
		97%	95%
**Diversity (Shannon-Wiener)**	**3 months**	3.17(±0.24)	3.10(±0.23)
	**10 months**	2.75(±0.28)	2.61(±0.28)
	**17 months**	3.48(±0.19)	3.39(±0.19)
	**NW**	3.28(±0.25)	3.18(±0.24)
	**TW**	3.16(±0.22)	3.09(±0.22)
	**TH**	3.34(±0.25)	3.28(±0.26)
	**Root endophyte**	3.16 (±0.21)	3.12 (0.20)
	**Rhizosphere**	3.51 (±0.21)	3.45 (±0.20)
	**TOTAL**	**3.64 (±0.17)**	**3.53 (±0.17)**
**Richness (Chao1)**	**3 months**	68(46–130)	47(36–80)
	**10 months**	37(30–63)	29(25–46)
	**17 months**	97(56–147)	57(47–94)
	**NW**	88(59–166)	69(48–126)
	**TW**	59(42–109)	49(37–86)
	**TH**	133(74–295)	101(61–211)
	**Root endophyte**	69 (50–123)	52 (42–85)
	**Rhizosphere**	145 (88–296)	121 (76–244)
	**TOTAL**	**134 (100–210)**	**98 (80–141)**

Moreover, the richness and diversity of the fungal community associated with sugarcane changed according to the sampling time; 17-month-old sugarcane plants had the highest diversity and richness indices. Plants 17 and 10 months old had significantly different diversity and richness indices (Table 1). In addition, impact of plant genotype (NW x TW) and crop management (TW x TH) on fungal richness and diversity was not observed.

The AMOVA allowed an assessment with a defined statistical probability of how much of the variation in the fungal community associated with sugarcane is due to genetically modified sugarcane planting (genotype effects), weed management (management effects), sampling time or isolation site (root endophyte or rhizosphere). Analysis of the results showed that plant genotype, crop management and the fungal community did not have significant effects on fungal community (P>0.05). However, the plant growth stage caused significant variation, contributing to 3.3% of the total variation (P = 0.0035) (Table 2). This variation was mainly related to the root endophytic community (3.21%, P = 0.0079); other groups did not contribute significant variation (P>0.05) (Table 2). These data suggest that plant age affects the endophytic root community but not the rhizosphere community.

**Table 2:** Analysis of molecular variance (AMOVA) for different fungus populations according to the evaluated effect.

Effect	Fungus Group	% variation ^1^	*P* value^2^
**Plant Genotype (Non-transgenic *vs*. transgenic)**	Total sample	-0.61	0.7019 ^*n*.*s*.^
	Plants with 3 months	0.00	0.6673 ^*n*.*s*.^
	Plants with 10 months	-4.76	1.0000 ^*n*.*s*.^
	Plants with 17 months	-0.71	1.0000 ^*n*.*s*.^
	Root endophyte	-1.16	0.7972 ^*n*.*s*.^
	Rhizosphere fungus	-1.68	0.6976 ^*n*.*s*.^
**Crop management (weeded *vs*. herbicide)**	Total sample	2.88	0.3041 ^*n*.*s*.^
	Plants with 3 months	8.65	0.3394 ^*n*.*s*.^
	Plants with 10 months	6.78	0.3301 ^*n*.*s*.^
	Plants with 17 months	1.08	0.6706 ^*n*.*s*.^
	Root endophyte	0.56	0.2052 ^*n*.*s*.^
	Rhizosphere fungus	-2.85	1.0000 ^*n*.*s*.^
**Plant growth stage (3 months *vs*. 10 months *vs*. 17 months)**	Total sample	3.30	**0.0035**
	Non transgenic plants (NW)	3.40	0.1323 ^*n*.*s*.^
	Transgenic plants with weeded (TW)	7.80	0.1299 ^*n*.*s*.^
	Transgenic plants with herbicide (TH)	-0.05	0.5971 ^*n*.*s*.^
	Root endophyte	3.21	**0.0079**
	Rhizosphere fungus	2.71	0.0731 ^*n*.*s*.^
**Fungus community (root endophyte *vs*. Rhizosphere fungus)**	Total sample	1.76	0.0971 ^*n*.*s*.^
	Non transgenic plants (NW)	-0.04	0.3971 ^*n*.*s*.^
	Transgenic plants with weeded (TW)	0.60	0.3023 ^*n*.*s*.^
	Transgenic plants with herbicide (TH)	0.68	0.2988 ^*n*.*s*.^
	Plants with 3 months	1.24	0.2033 ^*n*.*s*.^
	Plants with 10 months	2.46	0.2943 ^*n*.*s*.^
	Plants with 17 months	1.36	0.2002 ^*n*.*s*.^

^**1**^ Percentage of variation between analyses groups;

^**2**^ *P*–probability of a variation component higher than the observed for the fixation index;

^***n*.*s***.^–non significantly values at the level of 5% probability.

### Correlation between genera and plant niche (root *vs*. rhizosphere)

The taxonomic similarities between fungi isolated from the roots (endophytes) and the rhizosphere were assessed by the Jaccard coefficient of similarity (J), considering the genera that occurred in each community. The value obtained (J = 0.343) indicated that these communities should have a greater number of genera that occurred infrequently than common genera.

**Fig 2:**
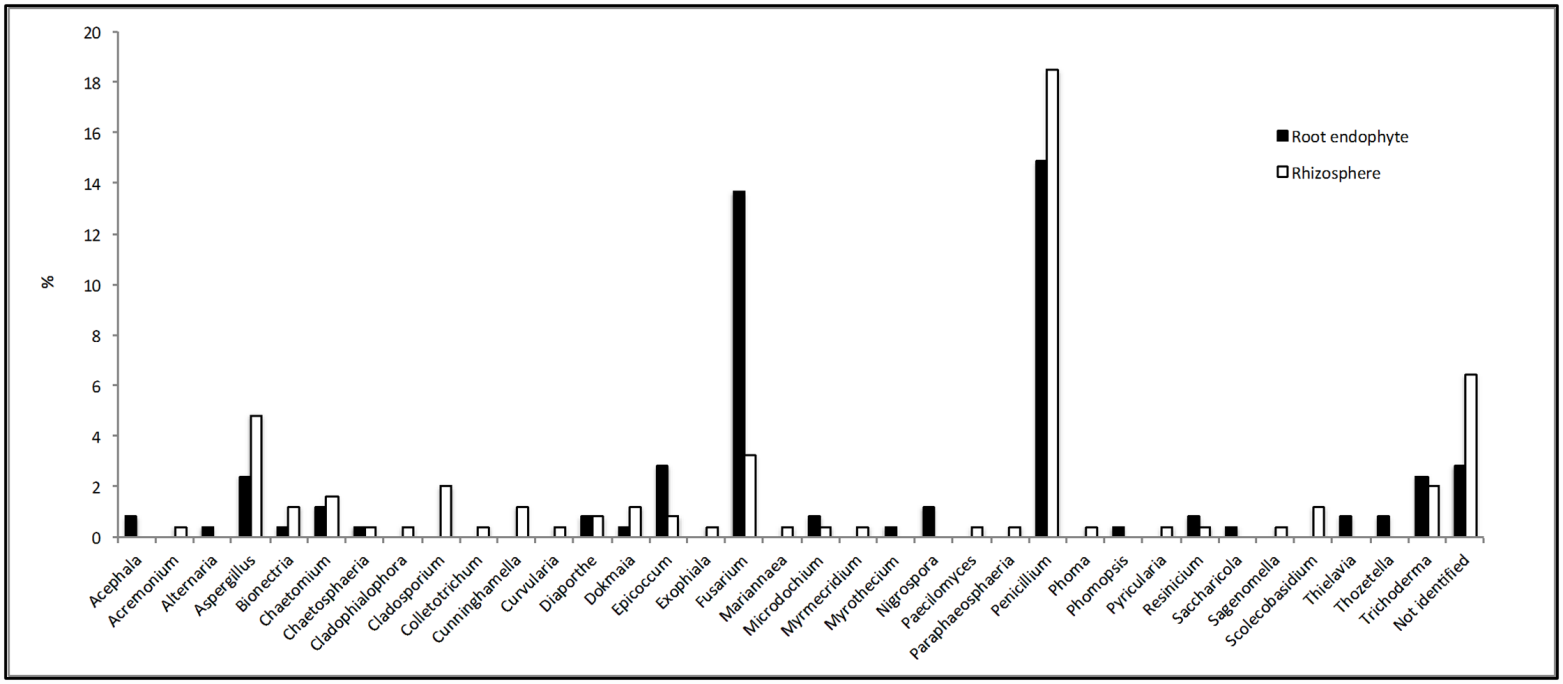
Percentage of the occurrence of each genus observed in the sugarcane fungal community and their relative proportions according to isolation location (rhizosphere or roots).

The proportion of rhizosphere and root endophytes in the community profile can be observed in Fig 2. The figure shows, for example, that 8 genera (*Acephala*, *Alternaria*, *Myrothecium*, *Nigrospora*, *Phomopsis*, *Saccharicola*, *Thielavia* and *Thozetella*) were observed only in roots and that 15 genera (*Acremonium*, *Cladophialophora*, *Cladosporium*, *Colletotrichum*, *Cunninghamella*, *Curvularia*, *Exophiala*, *Mariannaea*, *Myrmecridium*, *Paecilomyces*, *Paraphaeosphaeria*, *Phoma*, *Pyricularia*, *Sagenomella* and *Scolecobasidium*) were observed only in the rhizosphere. Moreover, some of the most frequent genera (such as *Aspergillus*, *Penicillium*, *Fusarium* and *Trichoderma*) were predominant in the roots or the rhizosphere. The genus *Aspergillus* was observed at a frequency two-fold higher in the rhizosphere than in the roots; on the other hand, 80% of the isolates identified as *Fusarium* were root endophytes (Fig 2), although this genus was also isolated from rhizosphere.

**Fig 3:**
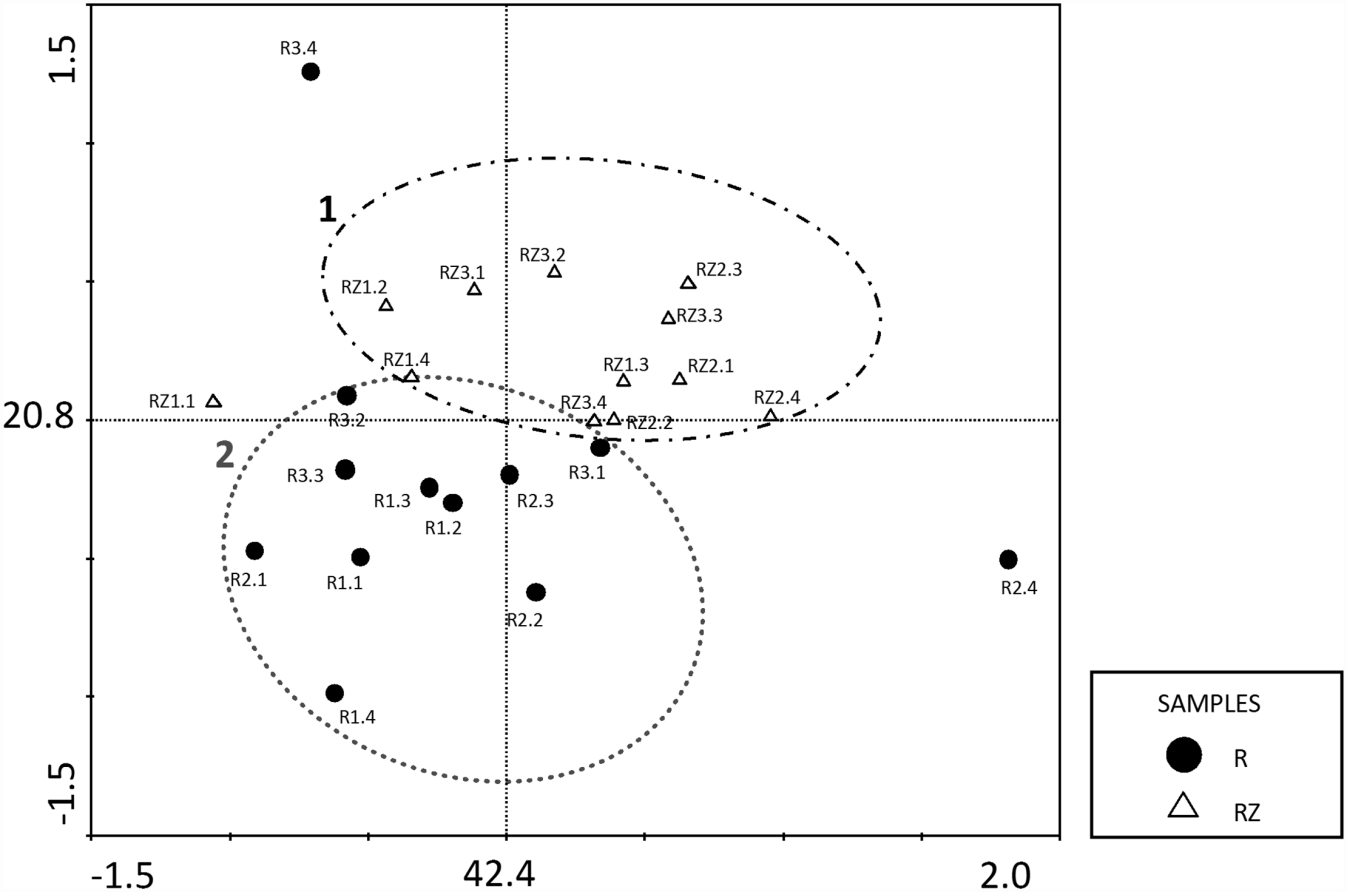
Principal Component Analysis (PCA) of the occurrence of the different fungus genera in the root endophytic community (● R) and the fungal rhizosphere community (Δ RZ). The values on both axes indicate the percentage of variance explained by sample distribution on each respective axis.

A principal component analysis (PCA) confirmed that the fungal communities in the roots and the rhizosphere were different (Fig 3). Although some fungi genera were shared by both inner roots and rhizosphere, the two principal components, which explain 63.2% of the variance, clustered the samples in two groups, being the first composed by rhizosphere community (RZ); and the second by endophytic community (R). These results indicated that the isolation site determined the variation of the frequency and the occurrence of such genera in the isolated fungal community.

### Genetic transformation of *T*. *virens* mediated by *A*. *tumefaciens*

The number of transformants that grew on selective medium was counted every 5 days for 30 days. The tests indicated that the optimal conditions for the transformation of *T*. *virens* fungus with *Agrobacterium* were the use of filter paper, inoculated with 10^7^ conidia mL^-1^, in PDA medium and co-cultivated for 48 h. Other conditions, such as two selection conditions were tested (*normal*–no addition of culture media to the membrane after the transfer to selective medium and *overlay–*the addition of culture media to the membrane) as well as two concentrations (200 and 400 μM) of the bacterial virulence inductor acetosyringone (AS).

The results showed that transformants were obtained under all tested conditions with similar transformation efficiencies. Overall, the average number of transformants obtained was 12 transformants per 10^7^ conidia. mL^-1^, and we obtained *T*. *virens* transformants with the chosen transformation conditions.

### Analysis of transformants

#### Mitotic stability

The mitotic stability of 20 random transformants was evaluated after 5 successive generations in non-selective medium. Of the transformants tested, 70% maintained hygromycin B resistance and green fluorescence emission (observed under FITC filter– 459 to 490 nm). Moreover, we did not observe sector emission in the evaluated transformants.

#### Molecular analysis

Eight transformants (T2, T4, T7, T9, T10, T15, T20, T22) resistant to hygromycin B were selected to confirm the presence of *gfp* gene by PCR using the glGFP5 and glGFP3 primers. All transformants tested and the plasmid pFAT-*gfp* (positive control) led to the amplification of a 700 pb fragment. The wild type strain *T*.*v*.223 was used as a negative control and did not allow amplification of this fragment.

Four transformants (T2, T7, T10 and T20) were randomly picked for Southern blot analysis from the eight transformants obtained to confirm the integration of the T-DNA genome and determine the number of copies inserted using a probe containing the *gfp* sequence (700 pb) (S4 Fig). The results confirmed that T-DNA was integrated into the genomes of all *T*. *virens* transformants and indicated the random occurrence of this event because different band sizes indicated different integration events. Transformants T20 and T2 had only one copy inserted, whereas the remaining transformants had more than one copy of T-DNA integrated into the genome. The wild type strain *T*.*v*.223 used as a negative control and the pFAT-*gfp* vector used as a positive control showed no bands and a 15 kb band, respectively.

#### Comparison of the transformants and the wild type *T*. *virens*

The mitotically stable T20 transformant that had a single copy inserted was selected for further analysis. The morphological and physiological similarities with the wild type strain *T*.*v*.**223** were evaluated. After 5 days of growth in PDA at 28°C, the morphological characteristics of both isolates were similar. Both showed weak and diffuse growth, with cottony mycelia and green conidia. The growth rates were similar; both grew approximately 2.7 cm per day in a Petri dish with PDA medium. Moreover, the *T*.*v*. T20 transformant and the wild type *T*.*v*.223 had similar conidial production (1.31 x 10^7^ conidia cm^-2^ and 1.71 x 10^7^ cm^-2^, respectively).

### Interaction of *T*. *virens* with the host plant

#### Reisolation of *T*. *virens* strain T20 from sugarcane plants

**Fig 4:**
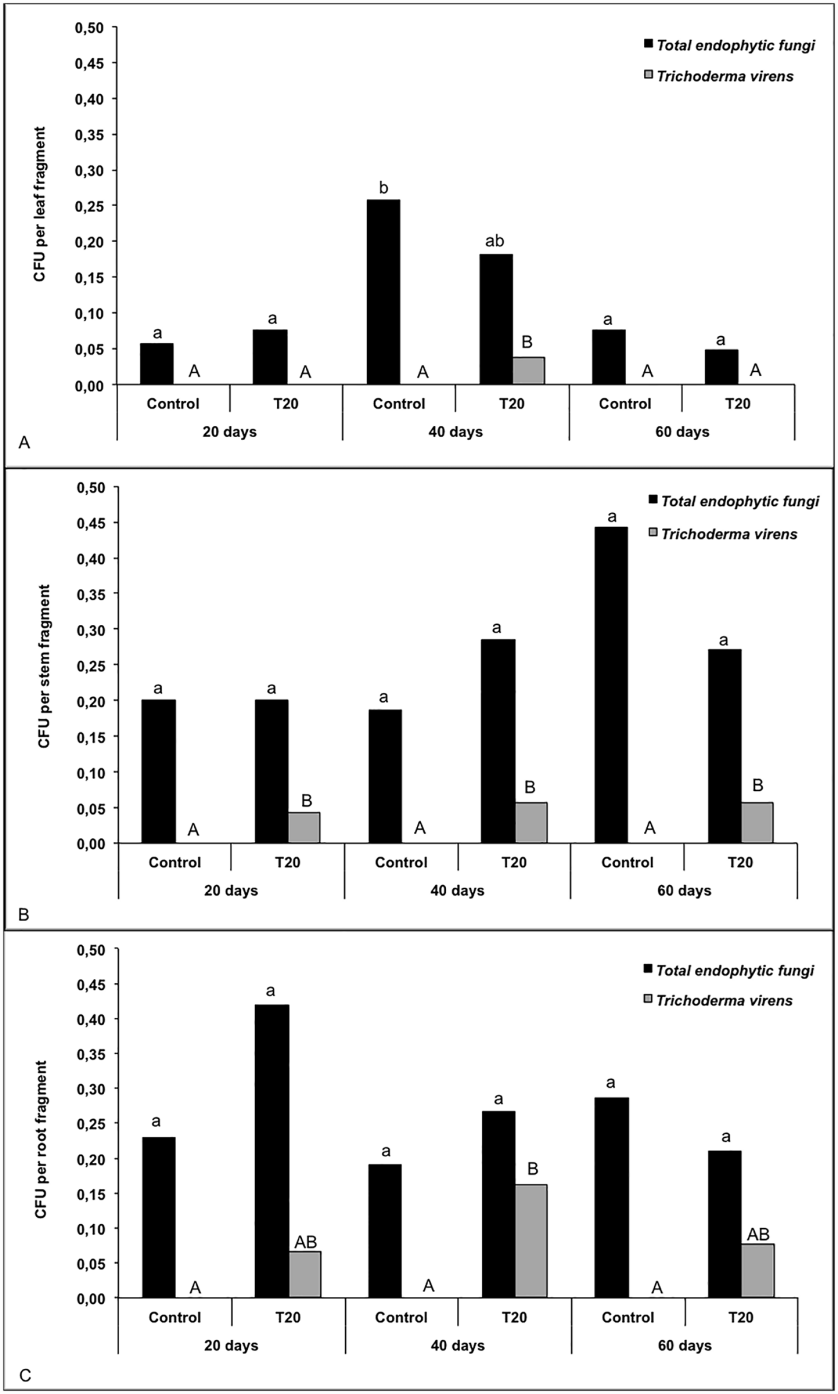
Isolation frequency of total endophytic fungi and *T*. *virens* strain T20 from leaves (a), stems (b) roots (c) of sugarcane plants at 20, 40 and 60 days. The same letter indicates that treatments are not significantly different according to Tukey’s test at 5% significance.

The results show that strain T20 endophytically colonized plant roots and stems but scarcely colonized plant aerial tissues. Root and stem colonization by T20 occurred at a low frequency (Fig 4) and did not lead to phenotypic changes in the plants. Moreover, the presence of T20 did not significantly affect the total frequency of endophytic fungi on the various plant tissues (Fig 4). Although this fungus is naturally found in sugarcane, in the present experiment *T*. *virens* could not be isolated from control (uninoculated) plants under any of the conditions tested (Fig 4); it was found only in inoculated plants.

The statistical analysis of the fungal reisolation frequency from sugarcane leaves showed that “*total endophytic fungi*” did not differ significantly between the inoculated and non-inoculated plants (S3 Table). However, a significant difference (P = 0.0418) was observed for the isolation of endophytic fungi in sugarcane plants at 20, 40 and 60 days; after forty days, plants showed a higher frequency of endophytic fungi.

A statistically significant difference for “*Total Trichoderma virens”* isolation was observed between the inoculated and uninoculated plants (P = 0.0415) and among the different plant ages (P = 0.0198), and their interaction was also significant (P = 0.0198). These differences are shown in Fig 4A, which shows that T20 was presented in inoculated plants only after 40 days. Similar results were observed in the stems and the roots. For both roots and shoots the “*Total endophytic fungus”* were not significantly different for either inoculated or non-inoculated plants and different isolation data. The variable “*Total Trichoderma virens”* showed significant differences between inoculated and non-inoculated plants, with a probability of P = 0.0222 for stems and P = 0.0005 for roots, indicating that that the frequency of *T*. *virens* reisolated from the inoculated plants was significantly higher than for the control plants because *T*. *virens* could not be isolated from the control plants (Fig 4B and 4C). Moreover, the reisolation frequency was not significantly different for different plant ages.

#### Monitoring of fungal colonization by fluorescence microscopy (FM) and scanning electronic microscopy (SEM)

**Fig 5:**
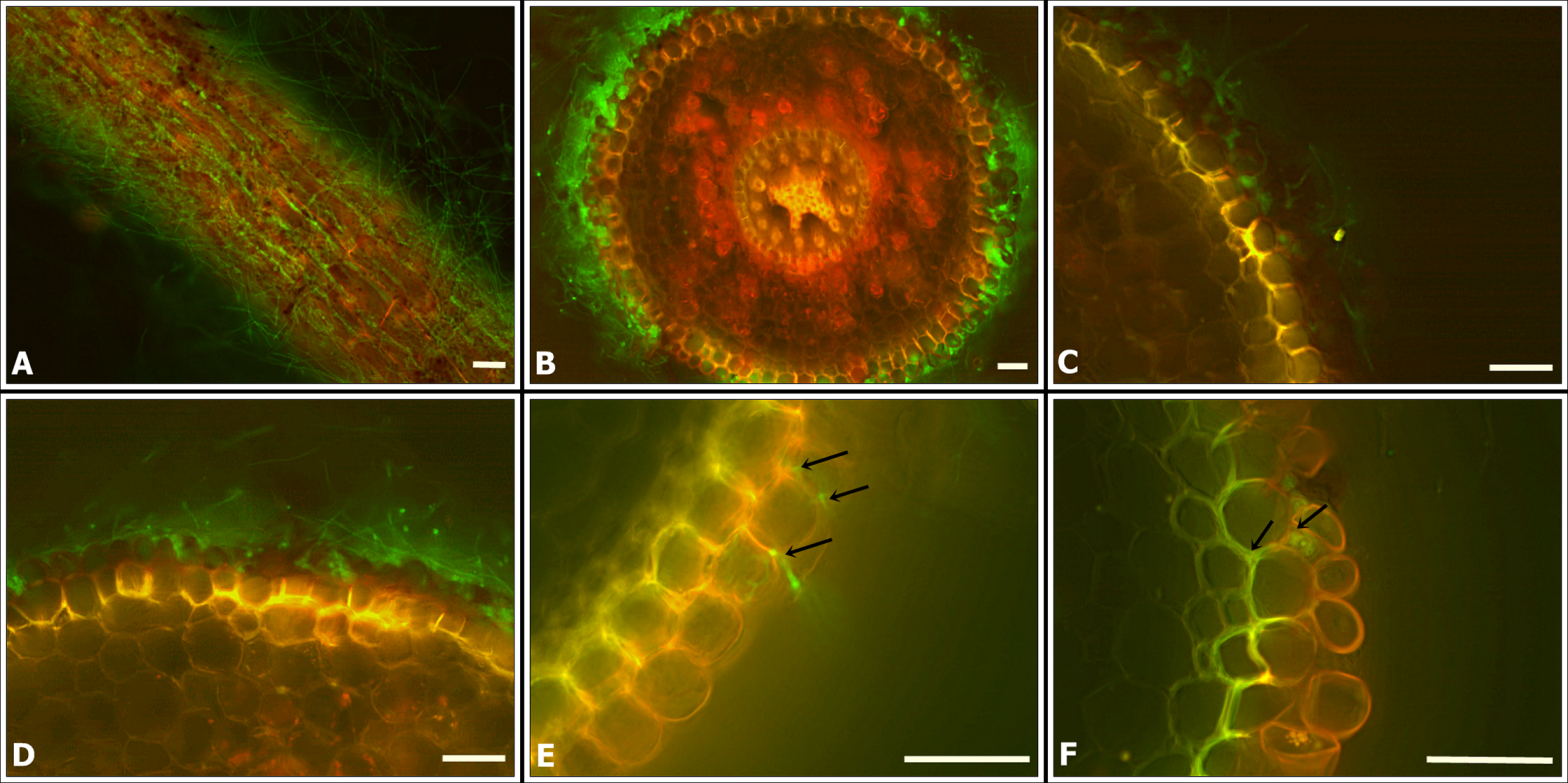
Optical fluorescence microscopy (OFM) with a FITC filter (459–490 nm), Rhodamine filter (546 nm) and an overlapped image with both filters, showing sugarcane root colonization (variety SP80-1842) by T20. **(A)** Sugarcane root with T20; **(B)** Cross section of sugarcane root with T20 **(C, D)** A longitudinal section of sugarcane colonized by T20 **(E, F)** Cross section of sugarcane root with T20; the arrows indicate possible fungal penetration in the outer layers of the root epidermis. Scale bars = 50 μm.

The superficial and internal colonization of sugarcane root tissue by *T*. *virens* fungus were observed by optical fluorescence microscopy (FM) and scanning electronic microscopy (SEM). The transformant T20 was selected for the microscopic analysis because it had a similar morphology, growth rate and sporulation as the wild type. Root tissue had an intense auto fluorescence, so two different filters were used: FITC (459–490 nm) and Rhodamine (546 nm). The fungus fluoresces only under the FITC filter, so root tissue could be distinguished from the fungus in an overlaid image. The conidia and hyphae of T20 transformant emit an intense green fluorescence (Fig 5), whereas the mycelium of the wild type did not. We were able to visualize dense mycelia in sugarcane roots inoculated with *T*.*v*. T20 (Fig 5A, 5B, 5C, 5D and 5F).

Conidial production was also observed in samples collected after 4 days. In addition to root surface colonization, in some cross sections, we were able to observe intercellular colonization of the outer root layers (Fig 5).

**Fig 6:**
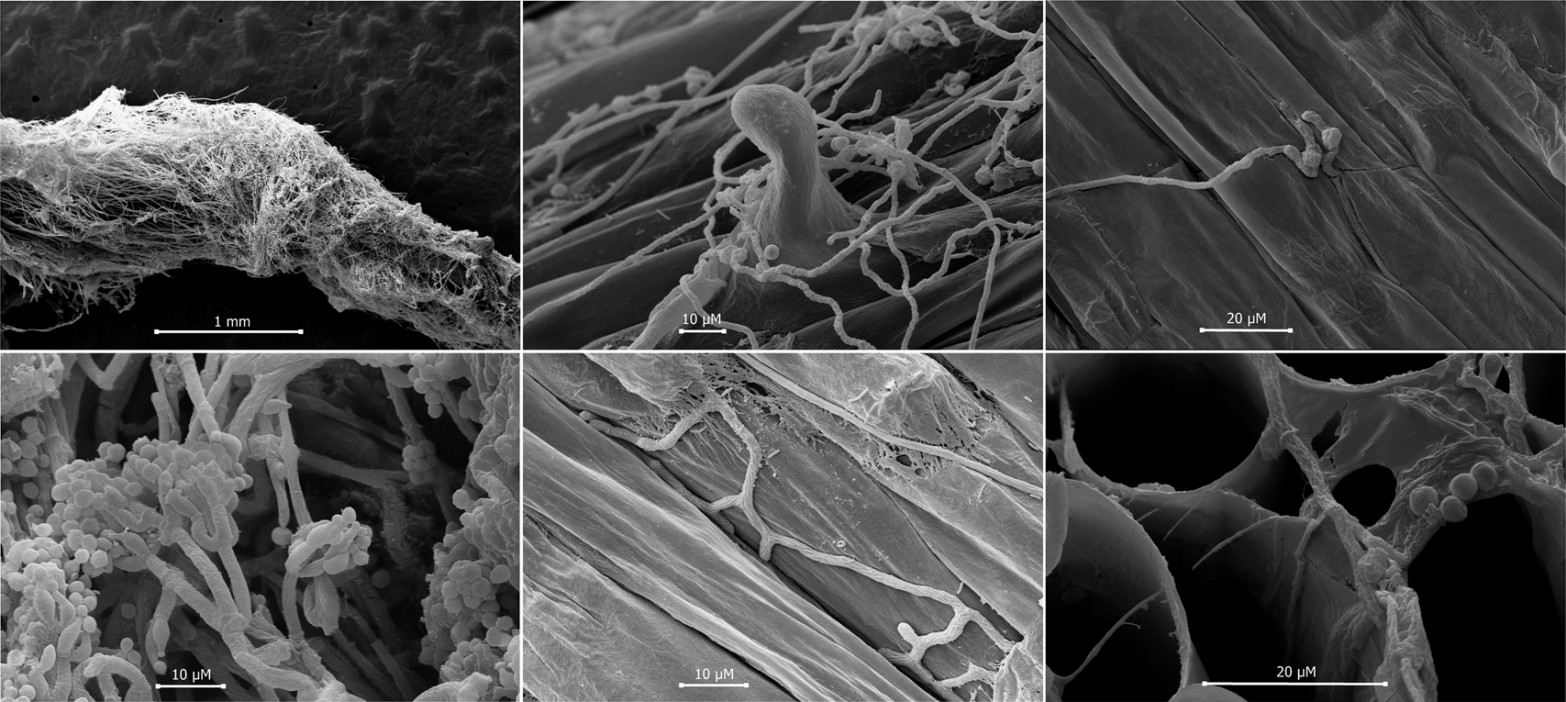
Scanning electronic microscopy images (SEM) showing sugarcane root colonization (variety SP80-1842) by *T*. *virens* strain T20. (**A, B**) Root surface colonization by T20; (**C**) *T*. *virens* conidia germination adhered to the plant host cuticle; (**D**) Asexual reproduction structure (conidiophore and conidia) of *T*. *virens* fungi; (**E**) Mycelia growth toward the cellular junctions and (**F**) Inner root segment showing the presence of fungal hyphae in the intercellular space. Scale bars are indicated in the figure.

Sugarcane root tissue infected with *T*. *virens* were also analyzed by SEM, which revealed interesting aspects of fungal behavior during plant colonization (Fig 6). First, we were able to observe the conidial germination on the root tissue surface and the elongation of hyphae adhered to the cuticle (Fig 6C). Moreover, the hyphae showed preferential growth toward the cellular junctions (Fig 6C and 6E). We could not see any appressorium or alteration of the plant surface in the presence of the fungus. Seven days after conidial inoculation, we were able to visualize dense colonization on the root surface, hyphae ramification, conidiospore formation and conidial production (Fig 6A, 6D and 6E). Observation of roots fractured with liquid nitrogen revealed the presence of fungal hyphae in the root tissue, in the intercellular space (Fig 6F).

#### The effect of *T*. *virens* on sugarcane growth

The results show that fungal inoculation did not result in any detectable phenotypical alteration and that all plants were healthy and vigorous with growth similar to the control. In fact, when the fresh and dry masses were compared, no significant differences were observed in shoots, roots or the entire plant in any treatment (Table 3). These results show that neither wild type nor transformant *T*. *virens* did not affect the growth of sugarcane under the conditions tested. Moreover, genetic alterations present in strain T20 apparently did not interfere with its interaction with sugarcane compared to the wild type strain.

**Table 3:** Effect of the inoculation of strains *T*.*v*.223 and *T*.*v* T20 on the fresh and dry weight accumulation on root and shoot of sugarcane plants (variety SP80-1842) cultivated for 60 days in greenhouse.

Treatment	Fresh mass (g) ^1^			Dry mass (g) ^1^		
	Root	Shoot	Total	Root	Shoot	Total
**SP80-1842 (control)**	5.12 ^**a**^	2.90 ^**a**^	8.02 ^**a**^	0.51 ^**a**^	0.83 ^**a**^	1.34 ^**a**^
**SP80-1842 + *T*.*v*. *strain* 223**	4.75 ^**a**^	2.53 ^**a**^	7.27 ^**a**^	0.43 ^**a**^	0.68 ^**a**^	1.12 ^**a**^
**SP80-1842 + *T*.*v*. strain T20**	4.66 ^**a**^	2.66 ^**a**^	7.33 ^**a**^	0.47 ^**a**^	0.69 ^**a**^	1.16 ^**a**^

^**1**^ Values are the average of 23 repetitions;

Treatments with the same letters do not statistically differ in Tukey test, at 1% of significance.

To confirm that the fungal inoculation of the soil was sufficient to promote sugarcane endophytic colonization and that the effects observed occurred in the presence of *T*. *virens*, some plants were sampled and endophytic fungi were isolated from sugarcane. Reisolation showed a low frequency of endophytic *Trichoderma* in the leaves and stems, similar to the previous reisolation results. *T*. *virens* was isolated only from the roots of inoculated plants, indicating that inoculation of the soil leads to the colonization of plant roots by *T*. *virens* but does not translocate to the shoot nor affect the plant biomass.

## Discussion

The indirect effect of genetically modified plants on ecosystem communities and functions are still being discussed [4, 54]. There is great concern about potential effects on non-target organisms such as mycorrhizae, rhizobium and other microorganisms that protect plants, as well as on phosphate solubilizing, decomposition and growth promotion.

The transgenic sugarcane plants in this study that are resistant to imazapyr (IMI-1) carry a plasmid containing a mutated wheat *AHAS* gene, which confers imazapyr resistance, that is under the control of a constitutive sugarcane promoter. The herbicide imazapyr inhibits the enzyme *AHAS*, which is responsible for the synthesis of some essential amino acids. This enzyme occurs in bacteria, fungi and plants, and has a similar amino acid sequence in these organisms, which suggests a common ancestor [55] and the potential that imazapyr could affect organisms other than weeds. Some studies have reported that nitrogen fertilizers affect the microbial community [56], as do imidazolinone-derived herbicides [20, 57], and transgenesis [20, 58, 59]; however, the results of this study indicated that neither the plant genotype (transgenic or non-transgenic) nor crop management (weeding or herbicide application) significantly changed the fungal community of cultivated sugarcane.

Interestingly, Stuart et al. [20] used the same field experiments as in the present study but evaluated only the first two growth stages (3 and 10 months) and observed an effect on the endophytic fungal community that colonized the leaves of the transgenic variety IMI-1 managed with imazapyr. They reported that the herbicide led to a rapid and transient alteration of the fungal community, whereas the transgenic variety displayed a slow and persistent change in the leaf fungal community [20]. Therefore, the effects of management and plant genotype seem to depend on the fungal community and probably on the plant growth stage.

However, similar to the present study, the lack of significant herbicide and/or genetically modified plants effects on the fungal community has been previously reported [5, 16, 60–62]. Indeed, the effect of genetic modification is low compared to that of other factors, such as the differences between plant varieties, which can be greater than those between genetically modified plants and their non-transgenic counterparts [63–65]. Therefore, it is likely that these studies did not detect any effect of plant genotype because other factors, such as the plant growth stage or crop management also significantly affected the microbial community, not mentioning other environmental factors, masking the lesser changes resulting from genetically modified plants.

Differently from genotype and crop management effects, the plant sampling time (3, 10 and 17 months) significantly affected the sugarcane endophytic fungal community, resulting in changes in the fungal frequency and the diversity and richness indices. This effect was also observed on endophyte in leaves by Stuart et al. [20]. Actually, the plant growth stage was previously reported to determine the microbial community structure, overcoming even the effects of genetically modified plants [11, 15, 66, 67]. Curiously though, the fungal community of the rhizosphere did not exhibit obvious changes in the parameters evaluated (fungal density, diversity and richness indices and molecular variance). Although culture dependent method could mask the presence of some important fungi, if the plant genotype or crop management had significant effect on these parameters, shifts in the fungi community would be observed.

The identification of the fungal community that colonizes the sugarcane roots and rhizosphere allowed us to calculate the diversity and richness indices. The results revealed that root endophytic fungi are less diverse than rhizosphere fungi, which we expected in view of the enormous fungal density in soil and the rhizosphere effect. The rhizosphere effect was demonstrated by Gomes et al. [68], who compared the DGGE profiles of the rhizosphere and soil fungal communities of maize and showed a significant increase in the relative abundance of fungi in the rhizosphere. Although the endophytic fungal diversity was lower than in the rhizosphere, the diversity index obtained (*H’* = 3,12) is similar to those reported in studies that evaluated endophytes from other host plants, such as *Guarea guidonia* [69].

The fungal community are structured by the isolation site [70–72], and it is likely that the community differs due to the different micro-environments in each plant tissue, soil type and rhizosphere. In overall, it was observed that the cultivable fungal community associated with sugarcane comprised at least 35 different genera, distributed in the phyla Ascomycota (96.0%), Zygomycota/Mucoromycotina (2.4%) and Basidiomycota (1.6%). The predominance of cultivable Ascomycota fungi associated with plants has been previously reported [73–77], whereas the lack of basidiomycetes is probably because methods that use plant fragments plated in growth broth do not favor their isolation [78]. In fact, several fungi cannot be retrieved with cultivable isolation methods, in this way the characterization of fungal communities by either dependent or independent cultivation methods can generate different taxonomic community profiles [58, 79], but both method can support conclusion about the impact assessment.

In Ascomycota population, two classes (Eurotiomycetes, Sordariomycetes) represented approximately 80% of the fungal community, and among these, 65.4% of the isolates belongs to *Penicillium*, *Fusarium*, *Aspergillus*, *Trichoderma* and *Epicoccum* genera, corroborating previous studies which showed that the fungal community associated to the host plant is composed by a great number of species, but are dominated by few species [80–82]. The genus *Epicoccum*, the fifth most frequent associated with sugarcane, was previously reported as an endophyte in banana [83] and apple trees [84], in the wheat rhizosphere [85] and in sugarcane leaves [86]. The genera *Penicillium*, *Fusarium*, *Aspergillus* and *Trichoderma* have been isolated both as endophytes [87–89] and from the rhizosphere of different host plants [90,91] and are usually the most frequent genera.

The data collected in this study together with the literature on the sugarcane fungal community revealed the presence of some ubiquitous fungal genera associated with the culture of this plant. For example, common genera in the rhizosphere fungal community were *Acremonium*, *Aspergillus*, *Cladosporium*, *Chaetomium*, *Cunninghamella*, *Curvularia*, *Fusarium*, *Penicillium*, *Trichoderma* and *Paecilomyces* [92], whereas the cosmopolitan endophytic fungi were *Aspergillus*, *Epicoccum*, *Fusarium*, *Penicillium* and *Trichoderma* [20]. The finding that 9.2% of the sugarcane fungal community could not be assigned using ITS sequencing to any known fungal genera opens the possibility of the discovery of novel fungal species because the great diversity of higher plants has long been considered the greatest reservoir of new fungi [93].

The genera *Penicillium* (teleomorph *Talaromyces* or *Eupenicillium*) and *Aspergillus* are ascomycota of Trichocomaceae family. Several species of this family have medical, industrial and biotechnological importance [94]. These genera can contain plant pathogens, generally not of economic importance but also harbour endophytes in several host plants [72, 87, 88, 89]. In this study, at least three different *Aspergillus* (*A*. *brasiliensis*, *A*. *fumigatus* and *A*. *terreus*) and *Penicillium* species (*P*. *pinophilum*, *E*. *javanicum* and *T*. *trachyspermus*) were observed. *Fusarium* genera (teleomorfe *Gibberella*) have also been reported to be plant pathogens and to cause great economic losses. In sugarcane, *F*. *verticillioides*, *F*. *moniforme* and *F*. *subglutinans* species have been reported as the casual agents of *Pokkah-boeng* and *Fusarium* rot, which remain latent until stress conditions trigger the plant disease [95, 96]. This genus has also been reported to contain endophytes, for example, *F*. *mangiferae* and *F*. *sterilihyphosum* in mango [97], *F*. *solani* in palm trees [98], *F*. cf. *avenaceum*, *F*. *decemcellulare* and *F*. *solani* in *Manilkara bidentata* [99] and *F*. *graminearum* in wheat [89]. Moreover, some endophytic strains of *F*. *oxysporum* have great potential as biological control agents, for example, in cucumber against the pathogen *Pythium ultimum* [100]. In this study, 42 isolates were classified as *Fusarium*, and *F*. *oxysporum* was the predominant species.

The genus *Trichoderma* (teleomorph *Hypocrea*) is known for rapid growth, its ability to use different substrates and resistance to toxic compounds [101] showing a great plasticity which features could increase the fitness during plant interaction. These fungi colonize soil, roots and leaves, and have a ubiquitous distribution, predominating in soils in various climates [102, 103]. They have also been reported as endophytes in cacao [104, 105], *Azadirachta indica* [88], banana [106] and fig trees [107]. Some species are of great economic importance due to their ability to produce enzymes and antibiotics, their potential as biological control agents and the capacity to induce plant growth [108,109]. Specifically, in sugarcane, cellulase production was tested in *T*. *viride* to deconstruct the lignocellulose biomass from *Saccharum spontaneum* by fermentation [110] and the production of ethanol directly from sugarcane bagasse by a recombinant *T*. *reesei* [111]. This species has been reported to be an aggressive mycoparasite, able to synthesize antibiotics, induce phytoalexin production and plant host resistance [36], as well as to produce endochitinases used for biocontrol [112] and, in some cases, to promote plant growth [113–114]. Because of these features, *T*. *virens*, one of the most abundant species that is not described as a toxigenic fungus, was chosen to be used in the studies to understand the interaction of this sugarcane plants and the fungi communities, allowing not only to study the impact of plant genotype, sampling time and crop management, but also how these fungi may interact with the host plant.

The first step to study the *Trichoderma-*sugarcane interaction involved the generation of hygromycin-resistant fungal mutants expressing GFP by AMT transformation which has been used for transformation of several fungi [21, 47, 48]. A high mitotic stability was observed in the mutants we generated, similar to reports of transformations using *gfp* gene with the *Agrobacterium* system in other fungal species [21, 49, 115].

The ability of *T*. *virens* to colonize sugarcane tissues endophytically was confirmed by reisolation of the T20 mutant strain, using the hygromycin resistance and the GFP expression to confirm the identity of the reisolated strain. The results showed that *T*. *virens* could colonize sugarcane endophytically and was found mainly in the plant roots, since it was in low frequency in leaves *T*. *virens* was only detected 40 days after inoculation, indicating that this fungus may not translocate to the shoot. This result was in agreement with previous observations in which *Trichoderma* were not isolated from sugarcane leaves [20] but from sugarcane roots (this study). Likewise, preferential root colonization has been reported for other *Trichoderma* species [116, 117]. In fact, different plant tissue preferences by an endophytic fungus have been shown to be related to specific conditions present in each plant organ [21, 24, 71]. Overall, sugarcane colonization by the *T*. *virens* T20 mutant occurred at a low frequency and did not lead to plant phenotypic alterations or disease signs and did not affect the total endophytic fungal frequency. Given that endophytic colonization can be affected by the inoculum type and by the plant growth substrate [104], perhaps the low frequency of the T20 mutant may have resulted from the conditions used for the interaction study. Interestingly, in this pot interaction study, the fungal frequency varied according to plant age as previously observed in the field study, corroborating the fact that plant growth stage may significantly affect the fungal community. In addition, it was observed that the inserted genes (*hph* and *gfp*) were maintained in the T20 mutant even after colonizing plant tissues in the pot interaction experiment, confirming the high genetic stability of this strain.

Specific aspects of the colonization of *T*. *virens* in sugarcane roots were explored by microscopy, in which OFM and SEM were used to monitor fungal colonization of superficial and inner tissues during plant growth. The results showed that this fungus colonized the root surface, forming a dense mycelial mass. In addition, the observation of root transverse sections revealed that *T*. *virens* also entered the host plant tissues and colonized the intercellular space of the outer layers of the root epidermis. Some *Trichoderma* strains have been reported to be able to colonize only specific parts of the roots [118], although rhizosphere strains are known to be able to colonize the entire root surfaces for several weeks [119] or months [120].

Generally, the penetration of *Trichoderma* spp. into plant root tissue is limited to the outer cell layers [118, 121] and does not lead to disease signs [116]. The first step of an endophytic colonization may occur when the fungus enters and forms specialized structures such as an apressorium, as observed in *Discula umbrinella* on the leaf surface [122] and in *Piriformospora indica* on the root surface [123]. Curiously, in the present work, we did not observe apressorium formation or the presence of any other specialized structures, but growth proceeded toward the cell junction, suggesting that *T*. *virens* may penetrate plant root tissues by other mechanisms. Endophytic fungi, for instance, can enter the host plant through natural openings such as stomata or between epidermal cells through the cuticle via the action of lytic enzymes, as has been reported for the fungus *Beauveria bassiana* [124] and *E*. *nigrum* [21], and which could also occur with *T*. *virens*.

In addition to being largely known as biological control agents, *Trichoderma* species are also known for other indirect effects in plants [116], including plant growth promotion and raising productivity [120, 125]. In this regard, the effect of the *T*. *virens* fungus in the host plant has been broadly studied in cotton [36], but to date, no such results have been reported for sugarcane. This study also evaluated the effects of *T*. *virens* on sugarcane growth and the dry mass of the shoots and the roots. The results showed that the presence of this fungus on the roots did not affect the phenotypical characteristics, plant growth or dry mass accumulation. In addition, *T*. *virens* T20 imposed similar effects on sugarcane plants compared to the wild type strain. Transgenic strains do not always maintain similar characteristics in their interactions with the plant host, and changes of the fungal life style (from mutualistic to parasitic) during the interaction with the plant have been reported for the endophytic fungus *Epichloë festucae* due to a mutation in one gene (*nox*A) [126].

The exploration of the intimate aspects of fungal-plant interactions allowed the confirmation of an endophytic relationship between *T*. *virens* and sugarcane, the determination of the colonization pattern and its phenotypic effects. *T*. *virens* could possibly be a biological control agent in the sugarcane fungal community, inhibiting phytophatogens by competition or parasitism, but this should be further investigated. Finally, this is the first study that reports the interaction between genetically modified sugarcane and the root endophytic and rhizospheric fungi communities. In addition, a large number of fungal species was isolated, generating a rich strains collection, which could be used in future studies that seeking to explore fungus-plant interactions and for the evaluation of potential fungal isolates that have biotechnological and agronomic potential.

## Supporting Information

S1 Fig Morphological diversity observed in fungal community associated to sugarcane. Fungal colonies grown in PDA broth at 28°C for 5–10 days. (a) *Epicoccum* sp., (b) *Penicillium* sp., (c) *Chaetomium* sp., (d) *Fusarium* sp., (e) *Trichoderma virens*, (f) Not identified root endophyte fungi, (g) *Eupenicillium javanicum*, (h) *Acremonium* sp., (i) *Talaromyces trachyspermus*, (j) *Aspergillus niger*, (k) *Penicillium* sp., (l) *Epicoccum nigrum*, (m) *Mariannaea* sp., (n) *Fusarium* sp., (o) *Bionectria* sp., (p) *Myrmecridium schulzeri*.
(DOCX)

S2 Fig Phylogenetic tree built by *Neighbor-joining* method using Jukes and Cantor model for ITS1-5,8S-ITS2 sequence isolated from sugarcane root and rhizosphere. Reference sequences of GenBank were used and the sequences obtained in the present work were signaled with the following symbol ♦. *Bootstrap* values (n = 1000) lower than 50 are represented in nods. a) 70 fungi of *Penicillium* genera, using the ascomycota *Buergenerula spartinae* as outgroup. b) 39 fungi of *Fusarium* genera, using the ascomycota *Bionectria ochroleuca* as outgroup. c) 17 fungi of *Aspergillus* genera, using the ascomycota *Penicillium pinophilum* as outgroup. d) 11 fungi of *Trichoderma* genera, using the ascomycota *Hypomyces aurantius* as outgroup.
(DOCX)

S3 Fig Rarefaction analysis showing the number of expected phylotypes associated to sugarcane fungal community, for the different levels of similarity (100%, 99%, 97% and 95%) of ITS1-5,8S-ITS2 sequence. (a) Comparison between the expected number of phylotype for endophytic and root fungal population; (b) Number of phylotype expected for all fungal community associated to sugarcane (root endophyte + rhizosphere fungi).
(DOCX)

S4 Fig *Southern blot* hybridization of four randomly picked *T*. *virens gfp*-tagged strains. Genomic DNA of the isolates were digested with restriction enzyme *Eco*RI, which cuts T-DNA twice and do not cut *gfp* gene sequence, a electrophoresis were performed in 0.8% agarose gel, transferring to nylon membrane and hybridized with the 700 pb fragment (*gfp* gene) labeled with digoxigenine. Column 1 positive control (pFAT-gfp plasmid); column 2 is the negative control (Wild strain *T*.*v*.223); columns 3–6 are the transformed strains (T20, T10, T7 and T2). Visualized bands are indicated with arrow.
(DOCX)

S1 Table ANOVA for the treatments factors and growth period. (DOCX)

S2 Table Taxonomic classification of the 249 evaluated fungus at genera level, distributed according to the isolation place (root or rhizosphere, plant growth (3, 10 or 17 months) and treatment (NW, TW, TH). (DOCX)

S3 Table ANOVA for factors Strain (L) and isolation period (I) for both variable, total endophytic fungi and total *Trichoderma*. Experimental design totally randomized. Analysis were performed separately for each part of the plant (leave, stem and root).
(DOCX)
