# Supplementary material for: Diversity of Cultivated Fungi Associated with Conventional and Transgenic Sugarcane and the Interaction between Endophytic Trichoderma virens and the Host Plant
Source: PLoS One. 2016 Jul 14;11(7):e0158974. doi: 10.1371/journal.pone.0158974 (PMC4944904; doi:10.1371/journal.pone.0158974)
Supplement: S1 Fig — Fungal colonies grown in PDA broth at 28°C for 5–10 days. (a) Epicoccum sp., (b) Penicillium sp., (c) Chaetomium sp., (d) Fusarium sp., (e) Trichoderma virens, (f) Not identified root endophyte fungi, (g) Eupenicillium javanicum, (h) Acremonium sp., (i) Talaromyces trachyspermus, (j) Aspergillus niger, (k) Penicillium sp., (l) Epicoccum nigrum, (m) Mariannaea sp., (n) Fusarium sp., (o) Bionectria sp., (p) Myrmecridium schulzeri. (DOCX) [file pone.0158974.s001.docx]

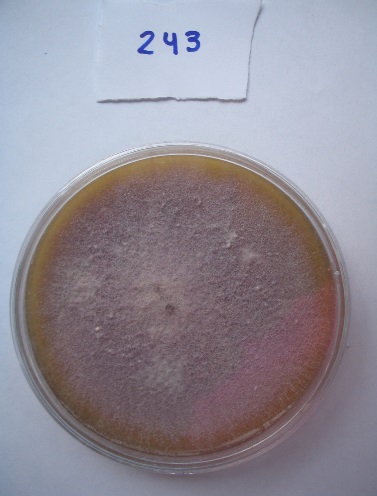

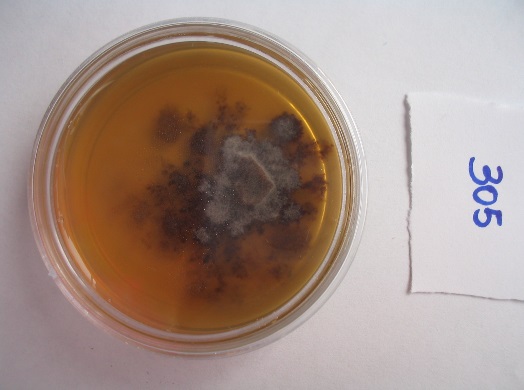

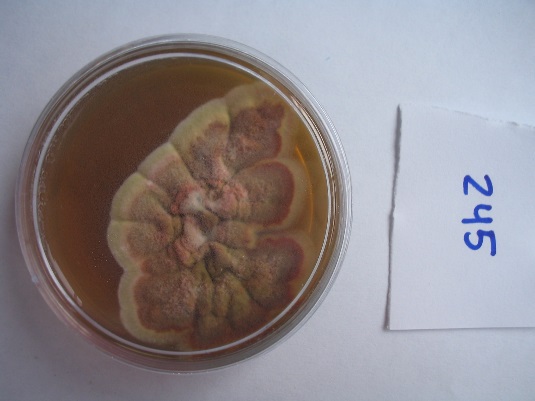

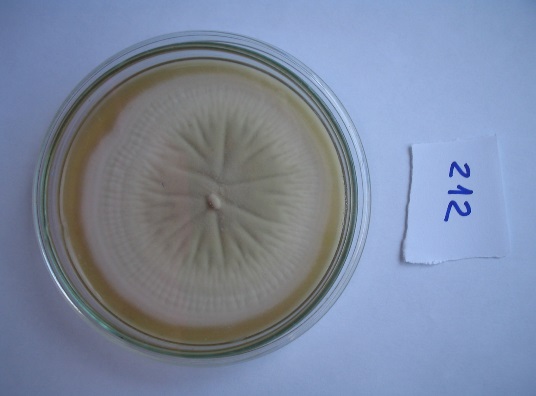

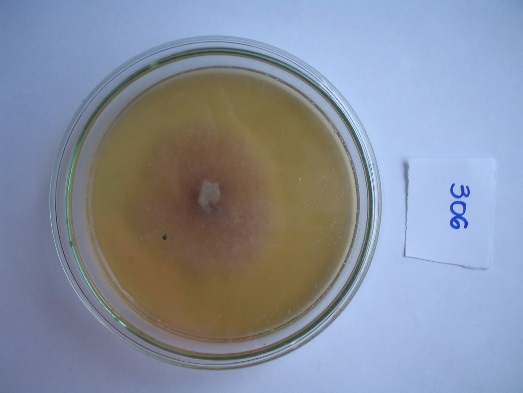

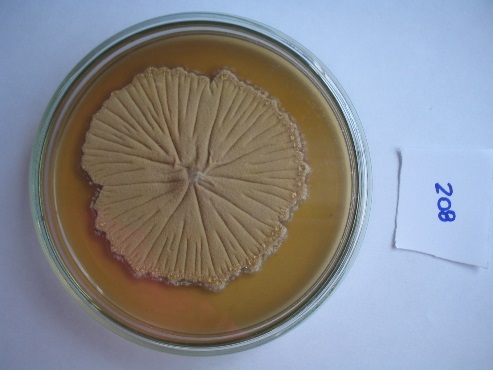

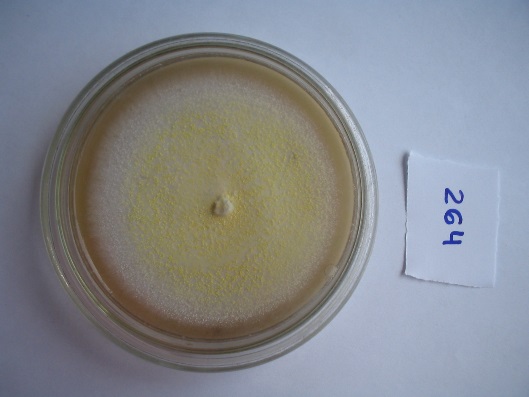

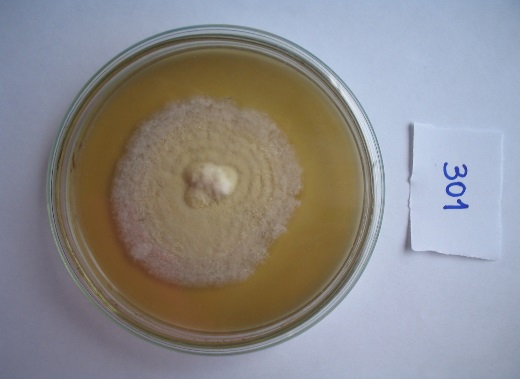

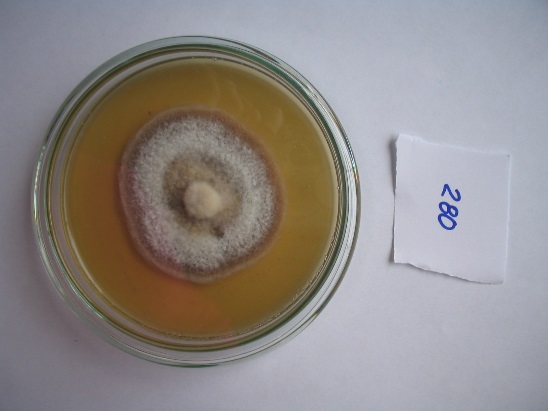

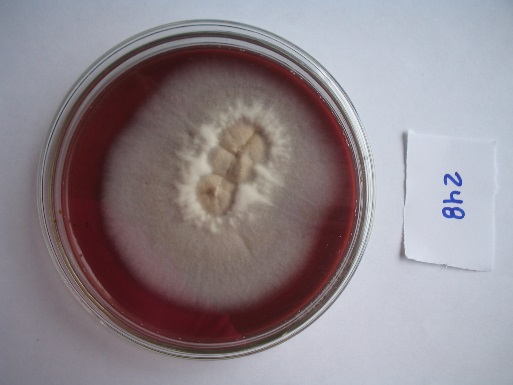

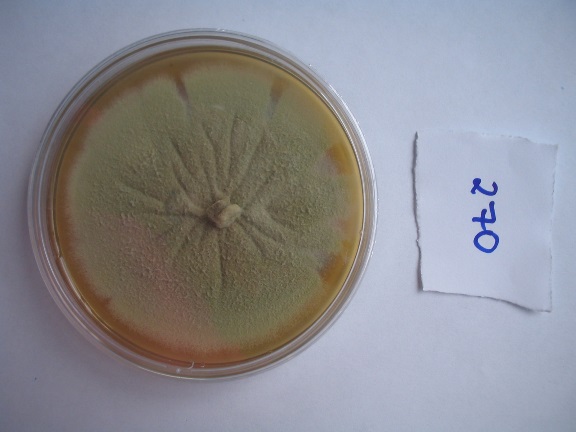

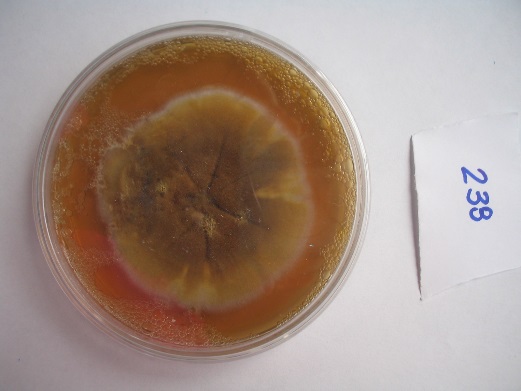

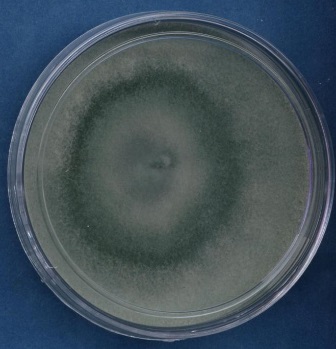

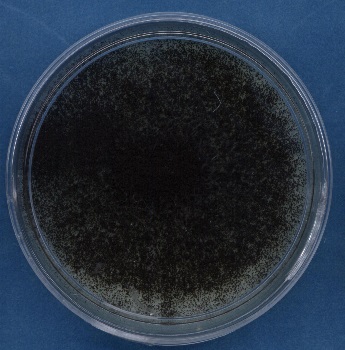

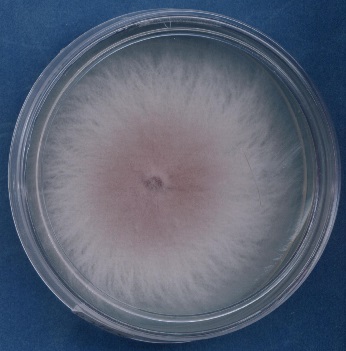


**a**

**b**

**c**

**d**

**e**

**f**

**g**

**i**

**j**


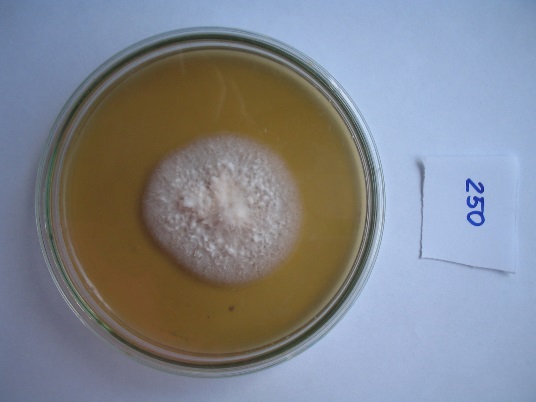


**h**

**k**

**l**

**m**

**n**

**o**

**p**

SM Figure 1 – Morphological diversity observed in fungal community associated to sugarcane. Fungal colonies grown in PDA broth at 28^o^ C for 5-10 days. (**a**) *Epicoccum* sp., (**b**) *Penicillium* sp., (**c**) *Chaetomium* sp., (**d**) *Fusarium* sp., (**e**) *Trichoderma virens*, (**f**) Not identified root endophyte fungi, (**g**) *Eupenicillium javanicum*, (**h**) *Acremonium* sp., (**i**) *Talaromyces trachyspermus*, (**j**) *Aspergillus niger,* (**k**) *Penicillium* sp., (**l**) *Epicoccum nigrum*, (**m**) *Mariannaea* sp., (**n**) *Fusarium* sp., (**o**) *Bionectria* sp., (**p**) *Myrmecridium schulzeri.*
