# Supplementary material for: Diversity of Cultivated Fungi Associated with Conventional and Transgenic Sugarcane and the Interaction between Endophytic Trichoderma virens and the Host Plant
Source: PLoS One. 2016 Jul 14;11(7):e0158974. doi: 10.1371/journal.pone.0158974 (PMC4944904; doi:10.1371/journal.pone.0158974)
Supplement: S2 Fig — Reference sequences of GenBank were used and the sequences obtained in the present work were signaled with the following symbol ♦. Bootstrap values (n = 1000) lower than 50 are represented in nods. a) 70 fungi of Penicillium genera, using the ascomycota Buergenerula spartinae as outgroup. b) 39 fungi of Fusarium genera, using the ascomycota Bionectria ochroleuca as outgroup. c) 17 fungi of Aspergillus genera, using the ascomycota Penicillium pinophilum as outgroup. d) 11 fungi of Trichoderma genera, using the ascomycota Hypomyces aurantius as outgroup. (DOCX) [file pone.0158974.s002.docx]

**Fig 2A**

Fig. 2B

Fig. 2.C

Fig. 2D

SM Figure 2 – Phylogenetic tree built by *Neighbor-joining* method using Jukes and Cantor model for ITS1-5,8S-ITS2 sequence isolated from sugarcane root and rhizosphere. Reference sequences of GenBank were used and the sequences obtained in the present work were signaled with the following symbol **♦**. *Bootstrap* values (n=1000) lower than 50 are represented in nods.

**a)** 70 fungi of *Penicillium* genera, using the ascomycota *Buergenerula spartinae* as outgroup;

**b)** 39 fungi of *Fusarium* genera, using the ascomycota *Bionectria ochroleuca* as outgroup.

**c)** 17 fungi of *Aspergillus* genera, using the ascomycota *Penicillium pinophilum* as outgroup;

**d)** 11 fungi of *Trichoderma* genera, using the ascomycota *Hypomyces aurantius* as outgroup.
