# Supplementary material for: Diversity of Cultivated Fungi Associated with Conventional and Transgenic Sugarcane and the Interaction between Endophytic Trichoderma virens and the Host Plant
Source: PLoS One. 2016 Jul 14;11(7):e0158974. doi: 10.1371/journal.pone.0158974 (PMC4944904; doi:10.1371/journal.pone.0158974)
Supplement: S3 Fig — (a) Comparison between the expected number of phylotype for endophytic and root fungal population; (b) Number of phylotype expected for all fungal community associated to sugarcane (root endophyte + rhizosphere fungi). (DOCX) [file pone.0158974.s003.docx]

SM Figure 3- Rarefaction analysis showing the number of expected phylotypes associated to sugarcane fungal community, for the different levels of similarity (100%, 99%, 97% and 95%) of ITS1-5,8S-ITS2 sequence. (**a**) Comparison between the expected number of phylotype for endophytic and root fungal population; (**b**) Number of phylotype expected for all fungal community associated to sugarcane (root endophyte + rhizosphere fungi).
