# Supplementary material for: Diversity of Cultivated Fungi Associated with Conventional and Transgenic Sugarcane and the Interaction between Endophytic Trichoderma virens and the Host Plant
Source: PLoS One. 2016 Jul 14;11(7):e0158974. doi: 10.1371/journal.pone.0158974 (PMC4944904; doi:10.1371/journal.pone.0158974)

SM Figure 4 - *Southern blot* hybridization of four randomly picked *T. virens gfp*-tagged strains. Genomic DNA of the isolates were digested with restriction enzyme *Eco*RI, which cuts T-DNA twice and do not cut *gfp* gene sequence, a electrophoresis were performed in 0.8% agarose gel, transferring to nylon membrane and hybridized with the 700 pb fragment (*gfp* gene) labeled with digoxigenine. Column **1** positive control (pFAT-gfp plasmid); column 2 is the negative control (Wild strain *T.v.***223**); columns **3-6** are the transformed strains (**T20,** **T10,** **T7** and **T2)**. Visualized bands are indicated with arrow.


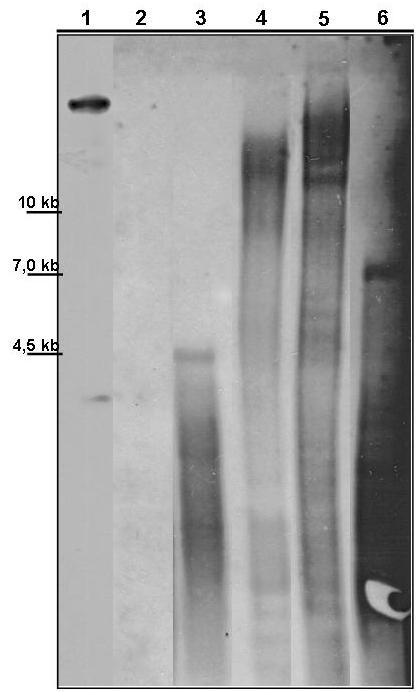

Supplement: S4 Fig — Genomic DNA of the isolates were digested with restriction enzyme EcoRI, which cuts T-DNA twice and do not cut gfp gene sequence, a electrophoresis were performed in 0.8% agarose gel, transferring to nylon membrane and hybridized with the 700 pb fragment (gfp gene) labeled with digoxigenine. Column 1 positive control (pFAT-gfp plasmid); column 2 is the negative control (Wild strain T.v.223); columns 3–6 are the transformed strains (T20, T10, T7 and T2). Visualized bands are indicated with arrow. (DOCX) [file pone.0158974.s004.docx]
