# Supplementary material for: Diversity of Cultivated Fungi Associated with Conventional and Transgenic Sugarcane and the Interaction between Endophytic Trichoderma virens and the Host Plant
Source: PLoS One. 2016 Jul 14;11(7):e0158974. doi: 10.1371/journal.pone.0158974 (PMC4944904; doi:10.1371/journal.pone.0158974)
Supplement: S1 Table — (DOCX) [file pone.0158974.s005.docx]

| SM Table 1 - ANOVA for the treatments factors and growth period. | | | | | |
| --- | --- | --- | --- | --- | --- |
| **A – Endophytic root fungus** | | | | | |
| **Variation Factor (VF)** | **DF** | **SQ** | **QM** | **F** | **P > F** |
| Blocs | 3 | 0.00979 | 0.00326 | 0.82 | 0.4963 ***^ns^*** |
| Treatments (T) | 2 | 0.01859 | 0.00929 | 2.33 | 0.1189 ***^ns^*** |
| Growth stage (G) | 2 | 0.03421 | 0.01710 | 4.29 | 0.0256 * |
| Interaction (T x G) | 4 | 0.05113 | 0.01278 | 3.21 | 0.0305 * |
| Error | 24 | 0.09572 | 0.00398 |  |  |
| Coefficient of variation (CV) = 5.22% | | | | | |
|  | | | | | |
| **B – Rhizosphere fungus** | | | | | |
| **Variation Factor (VF)** | **DF** | **SQ** | **QM** | **F** | **P > F** |
| Blocs | 3 | 209234 | 69744 | 1.44 | 0.2572 |
| Treatments (T) | 2 | 19263 | 9631 | 0.20 | 0.8216 |
| Growth stage (G) | 2 | 178217 | 89108 | 1.83 | 0.1816 |
| Interaction (T x G) | 4 | 147514 | 36878 | 0.76 | 0.5623 |
| Error | 24 | 1166459 | 48602 |  |  |
| Coefficient of variation (CV) = 33.59% | | | | | |
| * there is a significant difference between calculated F and tabled F (Pearson; Hartley, 1966) at 5% of probability.  Pearson, E. S., and H. O. Hartley (Edit.): Biometrika Tables for Statisticians Vol. I, 3. Auflage. University Press, Cambridge 1966. | | | | | |
