# Supplementary material for: Diversity of Cultivated Fungi Associated with Conventional and Transgenic Sugarcane and the Interaction between Endophytic Trichoderma virens and the Host Plant
Source: PLoS One. 2016 Jul 14;11(7):e0158974. doi: 10.1371/journal.pone.0158974 (PMC4944904; doi:10.1371/journal.pone.0158974)
Supplement: S2 Table — (DOCX) [file pone.0158974.s006.docx]

| SM Table 2– Taxonomic classification of the 249 evaluated fungus at genera level, distributed according to the isolation place (root or rhizosphere, plant growth (3, 10 or 17 months) and treatment (NW, TW, TH). | | | | | | | | | | | | | | | | | | | | | | | | | | | | | |  |
| --- | --- | --- | --- | --- | --- | --- | --- | --- | --- | --- | --- | --- | --- | --- | --- | --- | --- | --- | --- | --- | --- | --- | --- | --- | --- | --- | --- | --- | --- | --- |
| **Genera ^1^** | **Root Endophytes** | | | | | | | | | | | **Rhizosphere Fungus** | | | | | | | | | | | | | | **Total** | | **%** | |  |
|  | **3 months** | | | **10 months** | | | **17 months** | | | **Total** | | **3 months** | | | | **10 months** | | | | **17 months** | | | | **Total** | |  |  |  |  |  |
|  | **NW** | **TW** | **TH** | **NW** | **TW** | **TH** | **NW** | **TW** | **TH** | |  | | **NW** | **TW** | **TH** | | **NW** | **TW** | **TH** | | **NW** | **TW** | **TH** | |  | |  | |  | |
| *Acephala* | 0 | 0 | 0 | **1** | 0 | **1** | 0 | 0 | 0 | | **2** | | 0 | 0 | 0 | | 0 | 0 | 0 | | 0 | 0 | 0 | | **0** | | **2** | | **0.80** | |
| *Acremonium* | 0 | 0 | 0 | 0 | 0 | 0 | 0 | 0 | 0 | | **0** | | 0 | 0 | 0 | | 0 | 0 | 0 | | **1** | 0 | 0 | | **1** | | **1** | | **0.40** | |
| *Alternaria* | 0 | 0 | **1** | 0 | 0 | 0 | 0 | 0 | 0 | | **1** | | 0 | 0 | 0 | | 0 | 0 | 0 | | 0 | 0 | 0 | | **0** | | **1** | | **0.40** | |
| *Aspergillus* | 0 | 0 | **1** | 0 | 0 | 0 | **3** | **2** | 0 | | **6** | | 0 | 0 | **2** | | **1** | **1** | **1** | | **1** | **5** | **1** | | **12** | | **18** | | **7.23** | |
| *Bionectria* | 0 | 0 | 0 | 0 | 0 | 0 | 0 | **1** | 0 | | **1** | | **2** | 0 | 0 | | 0 | 0 | 0 | | 0 | **1** | 0 | | **3** | | **4** | | **1.61** | |
| *Chaetomium* | 0 | **1** | 0 | **1** | 0 | 0 | 0 | 0 | **1** | | **3** | | 0 | 0 | 0 | | **2** | 0 | **1** | | **1** | 0 | 0 | | **4** | | **7** | | **2.81** | |
| *Chaetosphaeria* | 0 | 0 | 0 | **1** | 0 | 0 | 0 | 0 | 0 | | **1** | | 0 | 0 | 0 | | **1** | 0 | 0 | | 0 | 0 | 0 | | **1** | | **2** | | **0.80** | |
| *Cladophialophora* | 0 | 0 | 0 | 0 | 0 | 0 | 0 | 0 | 0 | | **0** | | 0 | 0 | 0 | | 0 | 0 | 0 | | 0 | 0 | **1** | | **1** | | **1** | | **0.40** | |
| *Cladosporium* | 0 | 0 | 0 | 0 | 0 | 0 | 0 | 0 | 0 | | **0** | | 0 | **3** | **1** | | 0 | 0 | 0 | | 0 | **1** | 0 | | **5** | | **5** | | **2.01** | |
| *Colletotrichum* | 0 | 0 | 0 | 0 | 0 | 0 | 0 | 0 | 0 | | **0** | | 0 | 0 | **1** | | 0 | 0 | 0 | | 0 | 0 | 0 | | **1** | | **1** | | **0.40** | |
| *Cunninghamella* | 0 | 0 | 0 | 0 | 0 | 0 | 0 | 0 | 0 | | **0** | | **1** | 0 | **2** | | 0 | 0 | 0 | | 0 | 0 | 0 | | **3** | | **3** | | **1.20** | |
| *Curvularia* | 0 | 0 | 0 | 0 | 0 | 0 | 0 | 0 | 0 | | **0** | | 0 | **1** | 0 | | 0 | 0 | 0 | | 0 | 0 | 0 | | **1** | | **1** | | **0.40** | |
| *Diaporthe* | 0 | 0 | **1** | 0 | 0 | 0 | **1** | 0 | 0 | | **2** | | 0 | 0 | 0 | | 0 | 0 | 0 | | 0 | **2** | 0 | | **2** | | **4** | | **1.61** | |
| *Dokmaia* | 0 | 0 | **1** | 0 | 0 | 0 | 0 | 0 | 0 | | **1** | | 0 | **1** | 0 | | **1** | 0 | 0 | | **1** | 0 | 0 | | **3** | | **4** | | **1.61** | |
| *Epicoccum* | **3** | **2** | 0 | 0 | 0 | 0 | **1** | 0 | **1** | | **7** | | **1** | 0 | **1** | | 0 | 0 | 0 | | 0 | 0 | 0 | | **2** | | **9** | | **3.61** | |
| *Exophiala* | 0 | 0 | 0 | 0 | 0 | 0 | 0 | 0 | 0 | | **0** | | 0 | 0 | 0 | | 0 | 0 | **1** | | 0 | 0 | 0 | | **1** | | **1** | | **0.40** | |
| *Fusarium* | **4** | **4** | **8** | **3** | **4** | **4** | **3** | **3** | **1** | | **34** | | **1** | **2** | **2** | | 0 | 0 | **1** | | 0 | 0 | **2** | | **8** | | **42** | | **16.9** | |
| *Mariannaea* | 0 | 0 | 0 | 0 | 0 | 0 | 0 | 0 | 0 | | **0** | | 0 | 0 | 0 | | 0 | 0 | 0 | | **1** | 0 | 0 | | **1** | | **1** | | **0.40** | |
| *Microdochium* | 0 | 0 | 0 | **1** | **1** | 0 | 0 | 0 | 0 | | **2** | | 0 | 0 | 0 | | 0 | 0 | **1** | | 0 | 0 | 0 | | **1** | | **3** | | **1.20** | |
| *Myrmecridium* | 0 | 0 | 0 | 0 | 0 | 0 | 0 | 0 | 0 | | **0** | | 0 | 0 | 0 | | 0 | 0 | 0 | | **1** | 0 | 0 | | **1** | | **1** | | **0.40** | |
| *Myrothecium* | 0 | 0 | 0 | 0 | 0 | 0 | 0 | **1** | 0 | | **1** | | 0 | 0 | 0 | | 0 | 0 | 0 | | 0 | 0 | 0 | | **0** | | **1** | | **0.40** | |
| *Nigrospora* | 0 | 0 | 0 | 0 | **2** | 0 | 0 | **1** | 0 | | **3** | | 0 | 0 | 0 | | 0 | 0 | 0 | | 0 | 0 | 0 | | **0** | | **3** | | **1.20** | |
| *Paecilomyces* | 0 | 0 | 0 | 0 | 0 | 0 | 0 | 0 | 0 | | **0** | | 0 | **1** | 0 | | 0 | 0 | 0 | | 0 | 0 | 0 | | **1** | | **1** | | **0.40** | |
| *Paraphaeosphaeria* | 0 | 0 | 0 | 0 | 0 | 0 | 0 | 0 | 0 | | **0** | | 0 | 0 | **1** | | 0 | 0 | 0 | | 0 | 0 | 0 | | **1** | | **1** | | **0.40** | |
| *Penicillium* | **2** | **5** | **6** | **8** | **2** | **5** | **1** | **5** | **3** | | **37** | | **3** | **3** | **2** | | **11** | **7** | **4** | | **6** | **5** | **5** | | **46** | | **83** | | **33.3** | |
| *Phoma* | 0 | 0 | 0 | 0 | 0 | 0 | 0 | 0 | 0 | | **0** | | 0 | 0 | 0 | | 0 | 0 | 0 | | **1** | 0 | 0 | | **1** | | **1** | | **0.40** | |
| *Phomopsis* | 0 | 0 | 0 | 0 | 0 | 0 | 0 | **1** | 0 | | **1** | | 0 | 0 | 0 | | 0 | 0 | 0 | | 0 | 0 | 0 | | **0** | | **1** | | **0.40** | |
| *Pyricularia* | 0 | 0 | 0 | 0 | 0 | 0 | 0 | 0 | 0 | | **0** | | **1** | 0 | 0 | | 0 | 0 | 0 | | 0 | 0 | 0 | | **1** | | **1** | | **0.40** | |
| *Resinicium* | 0 | 0 | 0 | 0 | 0 | 0 | **1** | **1** | 0 | | **2** | | 0 | 0 | 0 | | 0 | 0 | 0 | | 0 | **1** | 0 | | **1** | | **3** | | **1.20** | |
| *Saccharicola* | 0 | 0 | 0 | 0 | **1** | 0 | 0 | 0 | 0 | | **1** | | 0 | 0 | 0 | | 0 | 0 | 0 | | 0 | 0 | 0 | | **0** | | **1** | | **0.40** | |
| *Sagenomella* | 0 | 0 | 0 | 0 | 0 | 0 | 0 | 0 | 0 | | **0** | | **1** | 0 | 0 | | 0 | 0 | 0 | | 0 | 0 | 0 | | **1** | | **1** | | **0.40** | |
| *Scolecobasidium* | 0 | 0 | 0 | 0 | 0 | 0 | 0 | 0 | 0 | | **0** | | 0 | 0 | **1** | | **1** | **1** | 0 | | 0 | 0 | 0 | | **3** | | **3** | | **1.20** | |
| *Thielavia* | **2** | 0 | 0 | 0 | 0 | 0 | 0 | 0 | 0 | | **2** | | 0 | 0 | 0 | | 0 | 0 | 0 | | 0 | 0 | 0 | | **0** | | **2** | | **0.80** | |
| *Thozetella* | 0 | 0 | 0 | 0 | **1** | 0 | 0 | **1** | 0 | | **2** | | 0 | 0 | 0 | | 0 | 0 | 0 | | 0 | 0 | 0 | | **0** | | **2** | | **0.80** | |
| *Trichoderma* | 0 | **1** | 0 | 0 | **1** | 0 | **2** | **2** | 0 | | **6** | | 0 | **2** | 0 | | 0 | 0 | 0 | | **1** | **1** | **1** | | **5** | | **11** | | **4.42** | |
| *Not identified* **^2^** | **1** | **1** | 0 | 0 | **1** | 0 | **2** | 0 | **2** | | **7** | | **2** | **1** | 0 | | **2** | **1** | **3** | | **4** | **3** | 0 | | **16** | | **23** | | **9.24** | |
| **^1^** In the cases that both phases (sexual and asexual) were observed they were grouped, this table refers to asexual fungus phase;  **^2^** ***Not identified*** – the fungus were not able to classified as a genera level. | | | | | | | | | | | | | | | | | | | | | | | | | | | | | |  |
