# Supplementary material for: Diversity of Cultivated Fungi Associated with Conventional and Transgenic Sugarcane and the Interaction between Endophytic Trichoderma virens and the Host Plant
Source: PLoS One. 2016 Jul 14;11(7):e0158974. doi: 10.1371/journal.pone.0158974 (PMC4944904; doi:10.1371/journal.pone.0158974)
Supplement: S3 Table — Experimental design totally randomized. Analysis were performed separately for each part of the plant (leave, stem and root). (DOCX) [file pone.0158974.s007.docx]

| SM Table 3 - ANOVA for factors Strain (L) and isolation period (I) for both variable, total endophytic fungi and total *Trichoderma*. Experimental design totally randomized. Analysis were performed separately for each part of the plant (leave, stem and root). | | | | | | | | | | | | |
| --- | --- | --- | --- | --- | --- | --- | --- | --- | --- | --- | --- | --- |
| **Organ** | **FV** | **Total endophytic fungi** | | | | |  | **Total *Trichoderma virens*** | | | | |
|  |  | **DF** | **SQ** | **QM** | **F** | **P-valor** |  | **DF** | **SQ** | **QM** | **F** | **P-valor** |
| **Leaves** | Treatments (T) | 5 | 0.0605 | 0.0121 | 1.60 ^ns^ | 0.1989 |  | 5 | 0.0028 | 0.0006 | 4.64 ** | 0.0042 |
|  | Strain (S) | 1 | 0.0018 | 0.0018 | 0.24 ^ns^ | 0.6291 |  | 1 | 0.0006 | 0.0006 | 4.64 * | 0.0415 |
|  | Isolation (I) | 2 | 0.0550 | 0.0275 | 3.63 * | 0.0418 |  | 2 | 0.0011 | 0.0006 | 4.64 * | 0.0198 |
|  | Interaction (LxI) | 2 | 0.0037 |  | 0.24 ^ns^ | 0.7884 |  | 2 | 0.0011 | 0.0006 | 4.64 * | 0.0198 |
|  | Error | 24 | 0.1818 | 0.0076 |  |  |  | 24 | 0.0029 | 0.0001 |  |  |
|  |  |  | | | | |  | | | | | |
| **Stem** | Treatments (T) | 5 | 0.0706 | 0.0141 | 1.09 ^ns^ | 0.3918 |  | 5 | 0.0092 | 0.0018 | 1.25 ^ns^ | 0.3183 |
|  | Strain (S) | 1 | 0.0011 | 0.0011 | 0.09 ^ns^ | 0.7697 |  | 1 | 0.0088 | 0.0088 | 5.97 * | 0.0222 |
|  | Isolation (I) | 2 | 0.0438 | 0.0219 | 1.69 ^ns^ | 0.2057 |  | 2 | 0.0002 | 0.0001 | 0.07 ^ns^ | 0.9359 |
|  | Interaction (LxI) | 2 | 0.0256 | 0.0128 | 0.99 ^ns^ | 0.3869 |  | 2 | 0.0002 | 0.0001 | 0.07 ^ns^ | 0.9359 |
|  | Error | 24 | 0.3110 | 0.0129 |  |  |  | 24 | 0.0355 | 0.0015 |  |  |
|  |  |  | | | | |  | | | | | |
| **Root** | Treatments (T) | 5 | 0.0515 | 0.0130 | 1.74 ^ns^ | 0.1639 |  | 5 | 0.0443 | 0.0088 | 4.42 ** | 0.0054 |
|  | Strain (S) | 1 | 0.0085 | 0.0085 | 1.44 ^ns^ | 0.2421 |  | 1 | 0.0326 | 0.0326 | 16.2 ** | 0.0005 |
|  | Isolation (I) | 2 | 0.0144 | 0.0072 | 1.22 ^ns^ | 0.3134 |  | 2 | 0.0058 | 0.0029 | 1.46 ^ns^ | 0.2513 |
|  | Interaction (LxI) | 2 | 0.0285 | 0.0142 | 2.41 ^ns^ | 0.1111 |  | 2 | 0.0058 | 0.0029 | 1.46 ^ns^ | 0.2513 |
|  | Error | 24 | 0.1421 | 0.0059 |  |  |  | 24 | 0.0482 | 0.0020 |  |  |
| **^ns^** – The values are not different at 5% f probability;  *, ** - The values are different at 5% and 1% of probability, respectively. | | | | | | | | | | | | |
